# Vδ1 T-cell subset appears to be responsive to PD-1 blockade therapy and is associated with survival in melanoma

**DOI:** 10.1136/jitc-2024-011224

**Published:** 2026-01-20

**Authors:** Nicola Herold, Jonas Bochem, Johanna Leyens, Svenja Wingerter, Stephan Forchhammer, Janine Spreuer, Malte Deseke, Can Yurttas, Paola Nocerino, Rita Antunes dos Reis, Teresa Amaral, Nikolaus B Wagner, Karolin Thiel, Daniel Soffel, Kristin Bieber, Patrick Terheyden, Daniela Wesch, Hans-Heinrich Oberg, Susanne Sebens, Manfred Claassen, Alfred Königsrainer, Claus Garbe, Graham Pawelec, Friedegund Meier, Markus W Löffler, Benjamin Weide, Immo Prinz, Sarina Ravens, Shahram Kordasti, Thomas Eigentler, Kilian Wistuba-Hamprecht

**Affiliations:** 1 Department of Dermatology, University Hospital Tübingen, Tübingen, Germany; 2 Section for Clinical Bioinformatics, Department of Internal Medicine I, University Hospital Tübingen, Tübingen, Germany; 3 M3 Research Center, University Hospital Tübingen, Tübingen, Germany; 4 Institut of Immunology, Medizinische Hochschule Hannover, Hannover, Germany; 5 Department of General, Visceral and Transplant Surgery, University Hospital Tübingen, Tübingen, Germany; 6 Systems Cancer Immunology, Comprehensive Cancer Centre, King’s College London, London, Germany; 7 Department of Dermatology, Venereology and Allergology, Kantonsspital St. Gallen, St. Gallen, Switzerland; 8 Department of Internal Medicine II, University Hospital Tübingen, Tübingen, Germany; 9 Department of Dermatology, University Hospital Schleswig-Holstein Lübeck Campus, Lübeck, Germany; 10 Institute of Immunology, University Medical Center Schleswig-Holstein, Campus Kiel, Christian-Albrechts University, Kiel, Germany; 11 Institute for Experimental Cancer Research, Kiel University and University Hospital Schleswig-Holstein, Campus Kiel, Kiel, Germany; 12 Department of Computer Science, University of Tübingen, Tübingen, Germany; 13 Institute for Bioinformatics and Medical Informatics, University of Tübingen, Tübingen, Germany; 14 Cluster of Excellence iFIT (EXC2180) 'Image-Guided and Functionally Instructed Tumor Therapies', University of Tübingen, Tübingen, Germany; 15 Department of Immunology, Institute for Cell Biology, University of Tübingen, Tübingen, Germany; 16 Health Sciences North Research Institute, Sudbury, Ontario, Canada; 17 Department of Dermatology, Faculty of Medicine and University Hospital Carl Gustav Carus, Technische Universität Dresden, Dresden, Germany; 18 Skin Cancer Center at the University Cancer Center Dresden and National Center for Tumor Diseases, Dresden, Germany; 19 Department of Clinical Pharmacology, University Hospital Tübingen, Tübingen, Germany; 20 Institute of Systems Immunology, Hamburg Center for Translational Immunology (HCTI), Hamburg, Germany; 21 Department of Clinical and Molecular Sciences, Università Politecnica delle Marche, Ancona, Italy; 22 Haematology Department, Guy's Hospital, London, UK; 23 Department of Dermatology, Venereology, and Allergology, Charité – Universitätsmedizin Berlin, corporate member of Freie Universität Berlin and Humboldt-Universität zu Berlin; Berlin, Berlin, Germany; 24 Skin Cancer Unit, German Cancer Research Center (DKFZ) Heidelberg, Heidelberg, Germany; 25 Department of Dermatology, Venereology and Allergology, University Medical Center Mannheim, Ruprecht-Karl University of Heidelberg, Mannheim, Germany

**Keywords:** T cell, Immune Checkpoint Inhibitor, Skin Cancer, Biomarker

## Abstract

**Background:**

Although most studies of anticancer T-cell immunity focus on αβ T cells, γδ T cells are attracting increasing attention due to their involvement in antitumor immune responses in various cancer entities, including melanoma. While immune checkpoint blockade (ICB) using the antagonistic programmed cell death protein 1 (PD-1) antibodies nivolumab and pembrolizumab significantly improved the survival of patients with melanoma with distant metastasis, prognosis remains poor. PD-1 is not only expressed by αβ T cells but also by γδ T cells, making this numerically minor population of unconventional T cells, whose role in melanoma is still elusive, a target of ICB.

**Methods:**

Here, we present a detailed γδ T-cell profiling study in late-stage melanoma at single-cell level using mass and polychromatic flow cytometry, T-cell receptor repertoire analyses and immunohistochemistry.

**Results:**

Our analyses link high frequencies of peripheral Vδ1 T cells before the start of anti-PD-1 therapy to a significantly reduced overall survival. In these patients, the Vδ1 compartment is dominated by a late-differentiated senescent-like phenotype that is presumably unresponsive to therapy. This phenotype is less prevalent at the tumor site and analysis of RNA sequencing data revealed that the abundance of Vδ1 T cells within the tumor was positively associated with survival.

**Conclusions:**

Our study suggests that Vδ1 T cells are associated with clinical outcomes, with a responsive subset expanding under ICB in patients where such a response remains possible. The observed clinical effects may be supported by the infiltration of these cells into the tumor, where they contribute to cancer immunosurveillance.

**WHAT IS ALREADY KNOWN ON THIS TOPIC**

- γδ T cells are an unconventional and numerically minor T-cell subset in peripheral blood, increasingly recognized for their role in antitumor immunity. The adaptive-like Vδ1 subset, enriched in barrier tissues, can exhibit potent cytotoxic activity but has also been associated with protumor functions in certain solid cancers. In melanoma, elevated peripheral Vδ1 frequencies correlate with poorer overall survival. While programmed cell death protein 1 (PD-1) immune checkpoint blockade (ICB) has significantly improved outcomes in melanoma via αβ T cells, the function of γδ T cells—also expressing PD-1—remains largely unexplored.

**WHAT THIS STUDY ADDS**

- This study demonstrates that elevated peripheral Vδ1 T-cell frequencies also prior to anti-PD-1 ICB are negatively associated with overall survival in patients with melanoma. This may be explained by an accumulation of late-differentiated senescent-like Vδ1 T cells in the periphery, likely resulting from chronic antigenic stimulation. In contrast, the intratumoral Vδ1 T-cell compartment consists of a lower proportion of this late-differentiated senescent-like phenotype and is associated with improved survival, suggesting a potential antitumor function. Furthermore, we identify Vδ1 T cells as direct targets of PD-1 blockade, which appears to promote the expansion of therapy-responsive Vδ1 clones.

**HOW THIS STUDY MIGHT AFFECT RESEARCH, PRACTICE OR POLICY**

- Our results highlight the predictive potential of Vδ1 T cells as a biomarker candidate in melanoma. Further in-depth profiling is needed to elucidate their functional heterogeneity and spatial organization within tumors, validate their biomarker capacity, and inform the development of novel therapeutic strategies, including the targeted exploitation of γδ T cells.

## Background

Immune checkpoint blockade (ICB), reinvigorating the antitumor T-cell response, induces durable remissions in advanced melanoma, although only in a minority of patients.^1^ Nowadays, therapeutic blockade of the immune checkpoint programmed cell death receptor 1 (PD-1) alone or in combination with targeting cytotoxic T-lymphocyte-associated protein-4 (CTLA-4) is standard-of-care treatment for metastatic melanoma. However, immune-related adverse events of treatment and disease-associated mortality resulting in a 5-year survival rate of around 50%^2^ underline the urgent need to decipher the mechanisms determining clinical responses.^3^

The inhibitory receptor PD-1 is expressed transiently on activated T cells, though persistent expression has been associated with T-cell dysfunction.^4 5^ Engagement of the ligands programmed cell death 1 ligand 1 (PD-L1) or PD-L2 results in impaired T-cell function, thereby regulating the immune response in order to maintain self-tolerance.^6^ This mechanism can be hijacked by cancers promoting immune evasion and providing the rationale for treatment with antagonistic antibodies.^7^ Besides αβ T cells, PD-1 is expressed on γδ T cells,^8^ making this non-classical T-cell population a further target of anti-PD-1 therapy. In addition to that, tumor cells can evade recognition by αβ T cells through loss of HLA class I. Yet, in colorectal cancer, the majority of patients with mutations in or a deficiency of β−2 microglobulin, an essential component of the HLA class I complex, benefit from therapy suggesting^9^ a participation of other immune cell subsets like γδ T cells that do not depend on peptide presentation by HLA. Indeed, de Vries *et al* recently demonstrated the contribution of γδ T cells to the response to PD-1 blockade in this setting.^10^ Furthermore, while loss of HLA class I compromises the clinical efficacy of anti-CTLA-4 therapy in melanoma, there was no association with response to PD-1 blockade.^11^

γδ T cells are, alongside αβ T cells and B cells, an evolutionarily conserved third subset carrying an antigen receptor assembled via somatic rearrangement.^12^ This unconventional, numerically minor T-cell subset uses a non-major histocompatibility complex (MHC)-restricted mode of antigen recognition and is considered an orchestrator of immune surveillance.^13^ The γδ T-cell compartment comprises distinct subsets with substantial functional diversity and is commonly delineated into innate-like Vγ9Vδ2 T cells and adaptive-like non-Vγ9Vδ2 T cells primarily encompassing Vδ1 T cells. Vγ9Vδ2 T cells are the predominant subset in peripheral blood, harbor a semi-invariant T cell receptor (TCR) repertoire^14^ and display phosphoantigen reactivity in a butyrophilin-dependent manner involving germline-encoded regions of the Vγ9 chain.^15 16^ Microbial infections or metabolic changes due, for instance, to a dysregulated mevalonate pathway in cancer cells leading to the accumulation of phosphorylated intermediates (termed phosphoantigens) are sensed by these cells.^17^ The Vδ1 subset is enriched in barrier tissues and is characterized by a private, highly diverse TCR repertoire typically showing stable clonotypic expansions.^18^ Ligands recognized by Vδ1 T cells include stress-induced antigens and MHC-like or MHC-related molecules. Atypical binding modes as well as the involvement of germline-encoded regions have been described.^19^,^21^ In addition to their somatically rearranged TCR, γδ T cells express natural cytotoxicity receptors and natural killer receptors.^22^ Both subsets show functional plasticity and a diverse array of antitumor functions has been documented, ranging from direct tumor killing via TRAIL or FASL and cytotoxic effector functions to antibody-dependent cellular cytotoxicity, antigen presentation capacity and interaction with B cells.^23^ However, also tumor-promoting effects mediated, inter alia, by interleukin (IL)-17, IL-4 and galectins have been reported.^23 24^

γδ T cells infiltrate primary melanomas as well as metastases.^25^ While higher proportions of intratumoral γδ T cells were linked to prolonged overall survival (OS),^26^ high frequencies of circulating Vδ1 T cells were negatively associated with survival in cutaneous melanoma.^27^ Knowledge regarding the role of PD-1 and the impact of ICB on γδ T cells, especially with respect to the Vδ1 subset, is limited. PD-1 is transiently upregulated on γδ T cells on stimulation,^28 29^ and PD-1 expression has been described on γδ T cells residing at the tumor site.^30^,^33^ In vitro studies demonstrated a diminished cytotoxic function of Vδ2 T cells in the presence of PD-L1, which could be reversed by an anti-PD-L1 monoclonal antibody (mAb), suggesting the presence of a functional PD-1/PD-L1 system in these cells.^34^ Furthermore, in an immunodeficient prostate cancer mouse model, anti-PD-1 treatment enhanced antitumor immunity of adoptively transferred Vγ9Vδ2 T cells.^28^ We have reported previously that high frequencies of Vδ1 T cells were associated with a late-differentiated phenotype and poor OS in patients with melanoma treated with ipilimumab.^35^

In the present study, we investigated the phenotype, functionality and TCR repertoire of peripheral blood and tumor-infiltrating γδ T cells in patients with late-stage melanoma receiving anti-PD-1 therapy, in order to further dissect their role and potential for exploitation in cancer immunotherapy.

## Methods

### Study design

The aim of this study was to investigate the role of γδ T cells in late-stage melanoma and the influence of anti-PD-1 therapy on this unconventional T-cell subset. Peripheral venous blood samples collected before the start of and under therapy were studied and correlated with clinical data. Via high-dimensional single-cell analysis, the immune signature of γδ T cells was explored in a discovery cohort. Flow cytometric analysis of two further independent patient cohorts online supplemental table S1 was performed to validate the findings. Furthermore, cytokine secretion and the proliferative capacity of γδ T cells were determined. The infiltration of γδ T cells into melanoma metastases was assessed by immunohistochemistry and flow cytometry. TCR sequencing was used to examine the TRD repertoire of peripheral blood and tumor-infiltrating γδ T cells.

###  Human samples

EDTA blood was obtained from healthy donors (HD) (median age: 61 (range 42–89), 61.8% male, 38.2% female) and patients with late-stage melanoma before the start of and under anti-PD-1 therapy. Patients receiving aminobisphosphonate therapy were excluded. Tumor specimens from surgically resected melanoma metastases and paired contemporaneous (−1 to +26 days after surgery) blood samples were collected. Pairwise analysis of individual blood and tumor samples across cellular subsets was performed to account for inter-individual variability and to control for potential batch effects, as the samples were processed and measured over a 6-year period. Bio-banked formalin-fixed paraffin-embedded (FFPE) blocks of melanoma metastases resected before the start of anti-PD-1 therapy were acquired.

### PBMC and TIL isolation

Peripheral blood mononuclear cells (PBMCs) were isolated from EDTA blood by Ficoll-Hypaque density gradient centrifugation (FicoLite-H, LINARIS), cryopreserved in RPMI-1640 medium (Sigma) containing 20% fetal calf serum (FCS) (Sigma) and 10% Dimethyl sulfoxide (DMSO) (SERVA) and stored in liquid nitrogen. Fresh tumor tissue from surgically resected metastases was finely minced and dissociated using the enzymes H and A from the human Tumor Dissociation Kit (Miltenyi) and a gentleMACS Dissociator (Miltenyi). The cell suspension was passed through a 100 µm cell strainer and washed with RPMI-1640. Freshly isolated tumor-infiltrating lymphocytes (TILs) were either immediately stained for fluorescence-activated cell sorting or cryopreserved as described above. Sorted γδ T cells were stored in RLT buffer (Qiagen) at −80°C for bulk TCR sequencing.

### Lymphocyte activation assay

Thawed, rested PBMCs were seeded in X Vivo 15 medium (Lonza) and stimulated with 20 ng/mL Phorbol 12-myristate 13-acetate (PMA) (Sigma) and 750 ng/mL ionomycin (Merck) or 10 µM zoledronate (Hexal) for 12 hours in the presence of brefeldin A (GolgiPlug, BD) and monensin (GolgiStop, BD). CD107a Pacific Blue (H4A3, BioLegend) was added directly to the culture medium. Subsequently, cells were stained for flow cytometry online supplemental figure S1. The polyfunctionality index was calculated using the following algorithm: $\sum_{i=0}^{n}F_{i}*\left(\frac{i}{n}\right)$, with *n* being the number of studied mediators and *F_i_* the frequency of cells expressing *i* mediators.^36^

### Proliferation assay

Cryopreserved PBMCs were thawed and rested in X-Vivo 15 medium before labeling with 5 µM CellTraceViolet (Invitrogen). Rested, labeled cells were stimulated with 5 µg/mL PHA-L (Roche) or 2.5 µg/mL zoledronate and 30 U/mL IL-2 (Proleukin S, Novartis) or 1 µg/mL immobilized anti-γδTCR mAb (IMMU510, Beckman) and 30 U/mL IL-2. After 5 days of culture, cells were stained for flow cytometry. Data was analyzed using FlowJo V.10.8.0 (BD, online supplemental figure S2) and ModFit LT (V.5.0, Verity Software House) was used to determine the proliferation index. PHA-L stimulation was used as a positive control and populations containing less than 100 events were excluded from proliferation analysis.

### Flow cytometry and fluorescence-activated cell sorting

Freshly isolated, frozen or cultured PBMCs and TILs were surface stained for lineage and differentiation markers. Cells were incubated with an Fc-receptor-blocking reagent (Gammunex, Grifols) and ethidium monoazide (EMA, Biotium) to label dead cells, or with LIVE/DEAD fixable red (Thermo Fisher Scientific) followed by Fc receptor blocking. An indirect staining approach using the therapeutic antibodies pembrolizumab and nivolumab for detection of PD-1 using anti-human IgG4-PE (Southern Biotech) was applied. ^37^ For staining of the γδ TCR, an unconjugated antibody in combination with a secondary antibody ^38^ or, to improve the stain index, a streptavidin-biotin-based protocol was used.^39^ Cell surface antigens were stained with antibodies directed against CD3 BV510 or Alexa700 (UCHT1 or OKT3), CD27 APC (O323), CD45 BV785 (HI30), CD45RA Pacific Blue (HI100), CD28 PE (CD28.2), CD57 APC or Pacific Blue (HCD57), Vδ2 PerCP (B6), PD-1 BV711 (EH12.2H7), Streptavidin-PE; all BioLegend. TCRγδ purified (11F2); BD. TCRγδ Biotin (11F2), Vδ1 FITC (REA173), Vδ2 APC (123R3); all Miltenyi. Vδ1 FITC (TS8.2), F(ab’)2-goat anti-mouse IgG Pacific Orange; all Invitrogen. For intracellular cytokine staining, cells were fixed and permeabilized with the Cytofix/Cytoperm Fixation/Permeabilization Solution Kit (BD) and stained with interferon (IFN)-γ PE-Cy7 (B27) and tumor necrosis factor (TNF) Alexa700 (Mab11) from BioLegend. Cells were acquired on an LSR II flow cytometer (BD) with FACSDiva software V.6.1.3 (BD). For fluorescence-activated cell sorting, a FACSAria II or FACSAria IIIu (BD) running on FACSDiva software V.8.0.1 or V.9.0.1 (BD) was used. Data were analyzed with FlowJo (gating strategies see online supplemental figure S1-S4) and populations with less than 100 events were excluded from further subset analysis.

### Mass cytometry

Cytometry by Time-Of-Flight (CyTOF) analysis followed established protocols.^40^ In brief, cryopreserved PBMC samples were thawed and 1.5–3×10^6^ cells were used for analysis. The applied antibody panel is summarized in online supplemental table S2. Rhodium was used as a dead cell marker and Fcγ receptors were blocked using Fc receptor blocking reagent (Kioving, Baxter). Surface antibody staining was performed in cell staining medium at room temperature. Samples were treated with a fixation/permeabilization kit (Thermo Fisher Scientific), following the manufacturer’s instructions, before intracellular antibody staining at 4°C. Samples were then resuspended in phosphate-buffered saline containing 4% paraformaldehyde (PFA) and stored overnight at 4°C. On the next day, each sample was individually incubated with 1x Iridium intercalator (Fluidigm) and buffered in H_2_O. Four-element EQ beads (Fluidigm) were added directly before acquisition on a Helios mass cytometer (Fluidigm) at the Flow Cytometry Core Facility, King’s College London. Per batch, up to five samples were sequentially acquired. FCS files were generated using the Helios software (Fluidigm) and the MATLAB tool from Finck *et al* was used to normalize for potential batch effects based on the four elements EQ beads.^41^ Quality control, data clean-up, concatenations, downsampling and visualization were done using FlowJo V.10.8.1 (BD, online supplemental figure S5. Only samples with >400 Vδ1 T-cell counts were considered for analyses. viSNE plots were calculated for data visualization, FlowSOM^42^ clustering was performed to identify cell subsets of interest and results were overlaid on the respective t-distributed stochastic neighbor embedding (t-SNE) plot using Cytobank (Beckmann Coulter). To visualize the cluster distributions of the individual patient samples, the cell counts per sample were downsampled to 433 cells (sample with the lowest counts). Next, concatenation of the Vδ1^high^ and Vδ1^low^ samples, followed by downsampling to 3,031 cells per group was performed for data visualization using density t-SNE plots. Heatmaps were generated using the mean of arcsinh(5) transformed marker expressions normalized to a mean of 0 and an SD of 1 per marker.

### TCR sequencing

RNA was isolated from sorted γδ T cells using the RNAeasy Micro Kit (Qiagen), followed by complementary DNA synthesis with the SuperScript reverse transcriptase (Invitrogen). For analysis of the TRD repertoire, CDR3 regions of the δ-chain were amplified via a multiplex PCR approach as described previously.^43^ Paired-end sequencing with 500 cycles was performed at the Illumina MiSeq platform.

### TCR repertoire analysis

Repertoire diversity represented by the Gini-Simpson Index and clonality represented by 1-Pielou’s Index were both calculated in Microsoft Excel. Pairwise similarity between repertoires described by the Morisita-Horn Index (MHI) was determined using the R package divo V.1.0.1. Circular tree maps were created with the R package packcircles V.0.3.4, Venn diagrams were generated using BioVenn.^44^ Shared and unique clonotypes of TILs were visualized using the R package UpSetR V.1.4.0.^45^

### Immunohistochemistry

Serial sections (3 µm) of FFPE tissue were stained with anti-CD3 (SP7; Thermo Scientific), anti-TCRγδ (H-41; Santa Cruz Biotechnology) or the corresponding isotype controls rabbit IgG (SP137; abcam), mouse IgG1 (11711; R&D Systems). Whole slide scans were acquired using a NanoZoomer V.2.0-HT digital slide scanner (Hamamatsu). H&E stained slide scans were examined by a histopathologist to identify tumor regions. Quantitative detection of CD3^+^ and TCRγδ^+^ cells was performed using QuPath V.0.2.3.^46^

### TCGA analysis

Clinical data and HTSeq-Counts from The Cancer Genome Atlas-skin cutaneous melanoma (TCGA-SKCM) project were acquired using R software V.4.2.0 and the R/Bioconductor package TCGAbiolinks V.2.24.3.^47^ To compile a cohort comparable to the patients analyzed in this study (described in online supplemental table S1) only patients with “AJCC pathologic stage” IV or IIIC were included. Patients with lymph nodes as the “site of resection or biopsy” were excluded. Normalization of HTSeq-Counts was performed using the variance stabilizing transformation included in the R package DESeq2 V.1.36.0.^48^ Based on the median TRDV1 expression patients were stratified into Vδ1^high^ and Vδ1^low^. “Days to death”, “days to last follow up” and “vital status” were used for survival analysis.

### Statistical analysis

Heatmaps were generated using the R package ComplexHeatmap V.2.12.1.^49^ Statistical significance was determined by Mann-Whitney test, Wilcoxon matched-pairs signed-rank test or log-rank test using SPSS Statistics V.28 (IBM), Prism V.5.04 (GraphPad) or the R packages survival V.3.4–0 and survminer V.0.4.9. P values were adjusted for multiple testing using the Benjamini-Hochberg procedure. A p value<0.05 was considered significant.

## Results

### Anti-PD-1 therapy modulates γδ T cells

**Figure 1:**
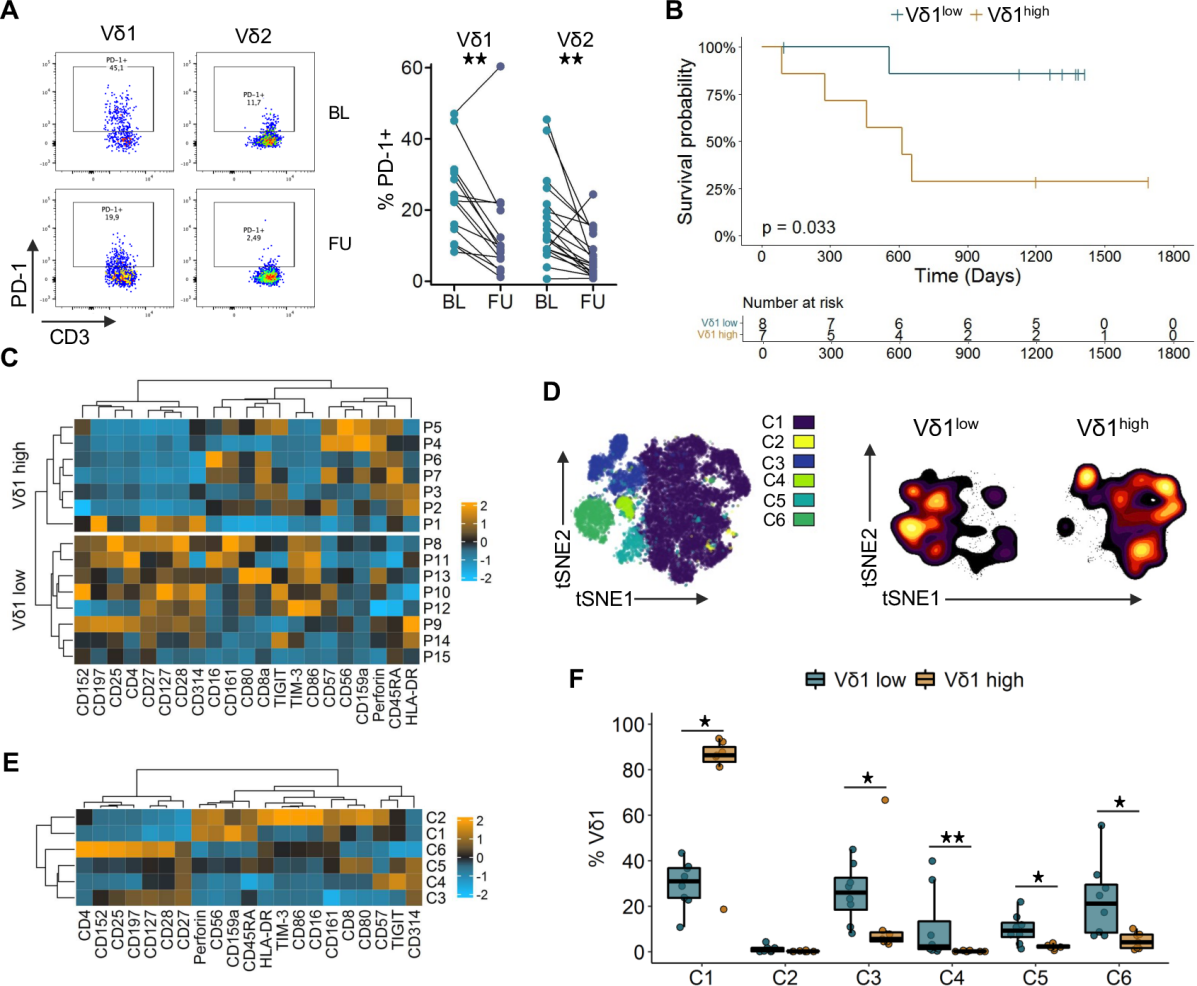
Vδ1 T-cell frequencies are associated with survival and different phenotypic profiles. (A) PD-1 expression on Vδ1 and Vδ2 T cells before the start of anti-PD-1 therapy at (BL; n=20–25) and at (FU; n=16–19) under therapy. (B) Overall survival of patients with late-stage melanoma dichotomized based on the BL median Vδ1 frequency (0.56%) determined by mass cytometry. Survival compared by log-rank test. (C) Heat map of phenotypic marker expression on Vδ1 T cells. Each row represents the BL sample of one patient, heatmaps are divided based on median Vδ1 frequency. (D) t-SNE visualization of Vδ1 T cells from BL and FU samples color coded by clusters identified using FlowSOM. Smoothed contour plots show BL samples of the patient groups with lower and higher than median Vδ1 frequencies. (E) Heat map depicting the phenotypic marker expression of the FlowSOM clusters identified in D. Only BL samples are shown. (F) Abundance of the identified FlowSOM clusters in the BL samples for the Vδ1low and Vδ1high group. Groups compared by Mann-Whitney U test, for paired analysis by Wilcoxon matched pairs signed-rank test *p<0.05, **p<0.01. Heat maps display marker expression, which was arcsinh-transformed and normalized to a mean of 0 and an SD of 1. Relative overexpression indicated in orange, relative underexpression in blue. BL, baseline; FU, follow-up; PD-1, programmed cell death receptor 1; t-SNE, t-distributed stochastic neighbor embedding.

Given that γδ T cells have received relatively limited attention in the context of ICB, we conducted a comprehensive analysis of peripheral blood and tumor tissue samples from patients with late-stage melanoma undergoing PD-1 blockade, aiming to obtain novel insights into the complex network of ICB modulation of cancer immunosurveillance. Expression of PD-1 on γδ T cells from patients with melanoma prior to as well as during anti-PD-1 therapy was analyzed by flow cytometry (figure 1A). PD-1 expression on patients’ peripheral blood γδ T cells at baseline (BL) before the start of therapy was comparable to HD with a median of 23.8% for Vδ1 and 12.0% for Vδ2 T cells online supplemental figure S6A. Using an indirect staining approach using therapeutic antibodies, we found a significant decrease in the frequency of PD-1^+^ Vδ1 (9.3%) and Vδ2 T cells (4.8%) early during anti-PD-1 immunotherapy (at a median of 46 days after starting therapy; figure 1A and online supplemental figure S6B). Furthermore, in follow-up (FU) samples collected during therapy, we detected therapeutic antibody bound to cell-surface PD-1 on Vδ1 T cells (online supplemental figure S6C). This confirms γδ T cells as direct targets of immune checkpoint blockade and suggests a modulation of both major γδ T-cell subsets.

### Mass cytometry reveals differences in Vδ1 profiles associated with survival

The phenotypic profile of γδ T cells in the blood of patients with late-stage melanoma online supplemental table S1 was characterized at high resolution using mass cytometry (CyTOF). Among all T cells, the median frequencies of Vδ1 and Vδ2 T cells were 0.56% and 0.88%, respectively, online supplemental figure S7. Such disproportionately high Vδ1 frequencies compared with Vδ2 have already been described by us as a characteristic of melanoma disease, compared with age-matched and sex-matched healthy individuals.^35^ Clinical data revealed a significant association of the abundance of Vδ1 T cells with OS (p=0.033). Above-median Vδ1 frequencies (Vδ1^high^) before the start of anti-PD-1 monotherapy were linked to poor OS (HR; 5.96), while patients with equal or lower than median Vδ1 frequencies (Vδ1^low^) had a prolonged OS (figure 1B). No association of survival with Vδ2 T-cell frequencies was observed in online supplemental figure S8. High-dimensional analysis showed that Vδ1 T cells strongly expressing CD25, CD27, CD28 or CD127 with a tendency to higher CD197 levels were enriched in the Vδ1^low^ group, whereas tendencies towards higher expression of CD57, CD45RA and perforin were more prominent in the Vδ1^high^ group (figure 1C and online supplemental figure S9). Of particular note is the patient P1 in the bottom row of the Vδ1^high^ heatmap with a phenotypic profile similar to the Vδ1^low^ group and having the lowest Vδ1 frequency (0.68%) within the Vδ1^high^ group: this patient had an above-median OS of 657 days. Cells from BL and FU samples were clustered unsupervised using FlowSOM and the resulting six clusters were projected onto a t-SNE visualization (figure 1D). Highlighting the BL samples for the Vδ1^low^ and Vδ1^high^ groups in a density visualization of the t-SNE plot revealed an opposing distribution (figure 1D); a similar pattern was observed under therapy online supplemental figure S11A. Cluster 3 and 6 showed the highest expression of CD27, CD28 and CD127, indicating early differentiated T cells (figure 1E, online supplemental figure S11). High expression of NKG2D (CD314) and intermediate levels of CD27 and CD127 are characteristic for clusters 4 and 5. These four clusters had a significantly higher abundance in patients of the Vδ1^low^ group (figure 1F). High levels of CD45RA and perforin together with low levels of CD27 and CD28, implying late-differentiated T cells, characterize clusters 1 and 2. Furthermore, the natural killer (NK) cell-related markers CD56, CD161 and NKG2A (CD159a) are upregulated in these two clusters. The Vδ1 T cells from Vδ1^high^ patients constitute the vast majority of cluster 1, while being almost absent from the other clusters, stressing the differences in the phenotypic profiles between long- and short-term survivors. This pattern suggests an accumulation of late-differentiated, potentially functionally impaired Vδ1 T cells in the periphery, rather than protumorigenic features, as a possible explanation for the observed negative association with clinical outcome. Comparison of the cluster frequencies revealed no distinct changes between BL and FU online supplemental figure S10B.

### Vδ1 T cells from short-term survivors are dominated by late-differentiated and replicative-senescent phenotypes

**Figure 2:**
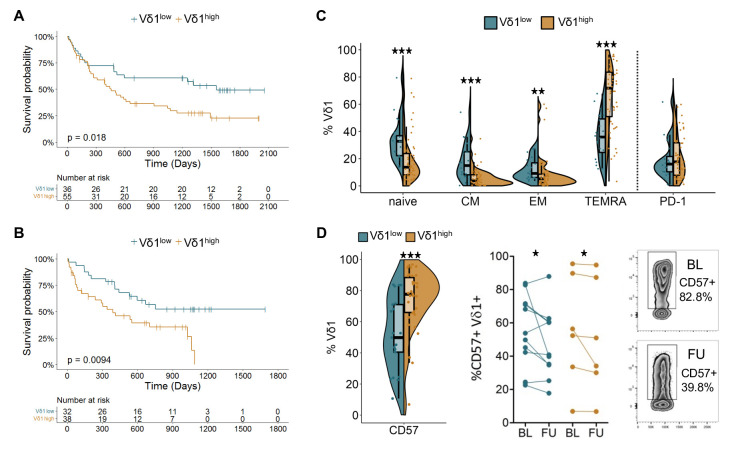
Flow cytometry reveals association of high Vδ1 T-cell frequencies with a TEMRA-like phenotype and high CD57 expression. (**A**) Overall survival of patients with late-stage melanoma in a validation cohort receiving anti-PD-1 monotherapy and (**B**) a second cohort treated either with anti-PD-1 alone or in combination with ipilimumab targeting CTLA-4. Patients were dichotomized based on the predetermined Vδ1 cut-off frequency of 0.56% among all T cells. Survival compared by log-rank test. (**C**) Distribution of memory differentiation profiles and PD-1 expression in the Vδ1^low^ and Vδ1^high^ group at BL for the cohort described in A (n=72). (**D**) Expression of CD57 at BL (n=56) and changes in expression under therapy (n=17) in the Vδ1^low^ and Vδ1^high^ group of the cohort described in B. Groups compared by Mann-Whitney U test, for paired analysis by Wilcoxon matched pairs signed-rank test. *p<0.05, **p<0.01, ***p<0.001. BL, baseline; CM, central memory CTLA-4, cytotoxic T-lymphocyte-associated protein-4; EM, effector memory; FU, follow-up; PD-1, programmed cell death receptor 1; TEMRA, terminally differentiated effector memory cells.

To validate these findings and further explore phenotypic variation, two independent cohorts of patients with late-stage melanoma online supplemental table S1 were investigated by polychromatic flow cytometry and stratified according to the previously determined Vδ1 cut-off frequency of 0.56%. Survival analysis confirmed the association of high Vδ1 frequencies with a reduced OS under anti-PD-1 monotherapy (figure 2A, p=0.018; HR: 1.92), as well as in patients receiving either anti-PD-1 monotherapy or combination treatment with ipilimumab targeting CTLA-4 (figure 2B, p=0.0094; HR: 2.34). Significantly higher levels of naive (CD27^+^CD45RA^+^), central memory (CD27^+^CD45RA^−^) and effector memory (CD27^−^CD45RA^−^) Vδ1 T cells were observed in Vδ1^low^ patients (figure 2C). In contrast to the rather balanced distribution of memory differentiation phenotypes in these patients, the Vδ1^high^ group was dominated by terminally differentiated effector memory cells re-expressing CD45RA (TEMRA; CD27^−^CD45RA^+^) at a median frequency of 71.8%. This is in line with the high abundance of the late-differentiated cluster 1 in Vδ1^high^ patients (figure 1F). Of note, no differences in PD-1 expression were observed between the two groups (figure 2C). While some patients exhibited changes in their memory differentiation profile from BL to FU, overall, a clear trend towards an increase or a decrease of a certain memory phenotype was lacking online supplemental figure S12. Expression of CD57, a putative marker for replicative senescence, on Vδ1 T cells at BL was comparable to HD online supplemental figure S13A, while patients’ Vδ2 T cells showed elevated percentages online supplemental figure S13B. Comparing the Vδ1^low^ to the Vδ1^high^ group, significantly higher percentages of CD57^+^ cells were detected in the latter (figure 2D). Under therapy, a decrease of CD57^+^ Vδ1 T cells was observed in both groups (figure 2D), whereas this was not seen for Vδ2 T cells online supplemental figure S13B. To rule out potential confounding factors influencing the observed association between Vδ1 T cells and patients’ OS, we performed both univariate and multivariate analyses using the combined data from both cohorts 2 and 3. High Vδ1 T-cell frequencies (p<0.001; HR: 2.18 online supplemental figure S14A, serum lactate dehydrogenase (LDH) ratio below the lower limit of normal (p=0.027; HR: 0.88), and M category (M1a or M1b; p=0.024; HR: 1.86) independently predicted overall survival online supplemental figure S14B; online supplemental figure table S3.

### Functionality of γδ T cells is altered in late-stage melanoma

**Figure 3:**
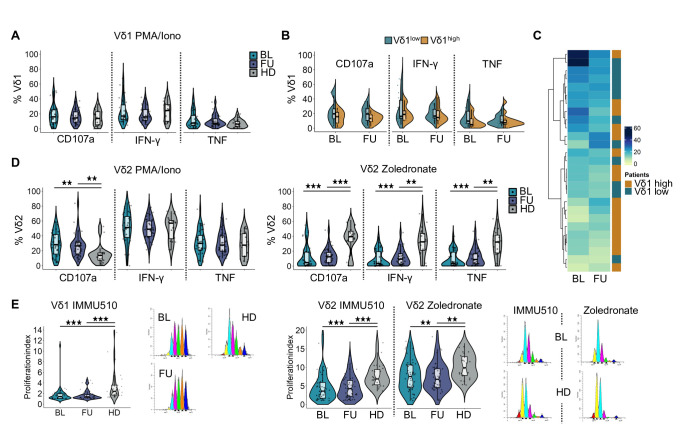
Functional analyses show altered cytokine expression and impaired proliferative capacity of γδ T cells in patients with melanoma. (**A**) Cytokine and CD107a expression in Vδ1 T cells after 12 hours stimulation of PBMCs from patients (BL: n=27; FU: n=30) and (HD; n=20) with PMA and ionomycin (Iono) in the presence of Golgi inhibitors. (**B**) Comparison of cytokine and CD107a expression between the Vδ1^low^ and Vδ1^high^ group. (**C**) Heatmap visualizing the polyfunctionality index (CD107a, IFN-γ, TNF) for Vδ1 T cells at BL and FU. Each row represents one patient. Heatmap divided by k-means clustering. (**D**) Cytokine and CD107a expression in Vδ2 T cells after 12 hours stimulation of PBMCs from patients and HD (n=20) with PMA and Iono or Zoledronate (BL: n=36; FU: n=32 to 34) in the presence of Golgi inhibitors. (**E**) Proliferation index for Vδ1 and (**F**) Vδ2 T cells determined by flow cytometry based on dye dilution. PBMCs from patients (BL: n=44 to 49; FU: n=37 to 46) and HD (n=25 to 27) were cultured for 5 days in the presence of an anti-TCRγδ antibody (IMMU510) or Zoledronate. Groups compared by Mann-Whitney U test. *p<0.05, **p<0.01, ***p<0.001. BL, baseline; FU, follow-up; HD, healthy donors; IFN, interferon; PBMC, peripheral blood mononuclear cell; TCR, T cell receptor; PMA, Phorbol 12-myristate 13-acetate; TNF, tumor necrosis factor.

We next sought to address whether the functional capacity of γδ T cells is altered in patients with melanoma and/or by anti-PD-1 therapy. Overall, low percentages of Vδ1 T cells expressed the degranulation marker CD107a and the cytokines IFN-γ and TNF in response to stimulation with PMA/ionomycin. Expression levels in patients at BL and FU were comparable to HD (figure 3A) and also similar between the Vδ1^low^ and Vδ1^high^ group (figure 3B). However, patients with low Vδ1 frequencies tended to have a higher polyfunctionality index,^36^ which takes the co-expression pattern of CD107a, IFN-γ and TNF into account (figure 3C and online supplemental figure S15). The functional response pattern of Vδ2 T cells differed from that of Vδ1 T cells and depended on the type of stimulus. While patients had a significantly higher proportion of CD107a-expressing cells at BL and FU in response to PMA/ionomycin when compared with HD, there were no differences in cytokine expression (figure 3D). In contrast, after stimulation with zoledronate, we observed lower levels of CD107a, IFN-γ and TNF in patients before and under therapy than in healthy individuals (figure 3D). This might be due to the different modes of action of these two stimuli. Whereas zoledronate leads to an accumulation of phosphoantigens, which indirectly activate the Vγ9Vδ2 T cells via interaction of their TCRs with butyrophilins, PMA and ionomycin mimic TCR signaling, but bypass the receptor itself. We also investigated proliferative potential using a dye dilution assay. The Vδ2 subset generally displayed a higher proliferation index than the Vδ1 subset (figure 3E and F). For both subsets, we observed significantly diminished proliferation in patients compared with HD after stimulation with an anti-TCRγδ antibody (IMMU510) or zoledronate.

### Increase in clonal diversity of the TRDV1 repertoire early under anti-PD-1 therapy

**Figure 4:**
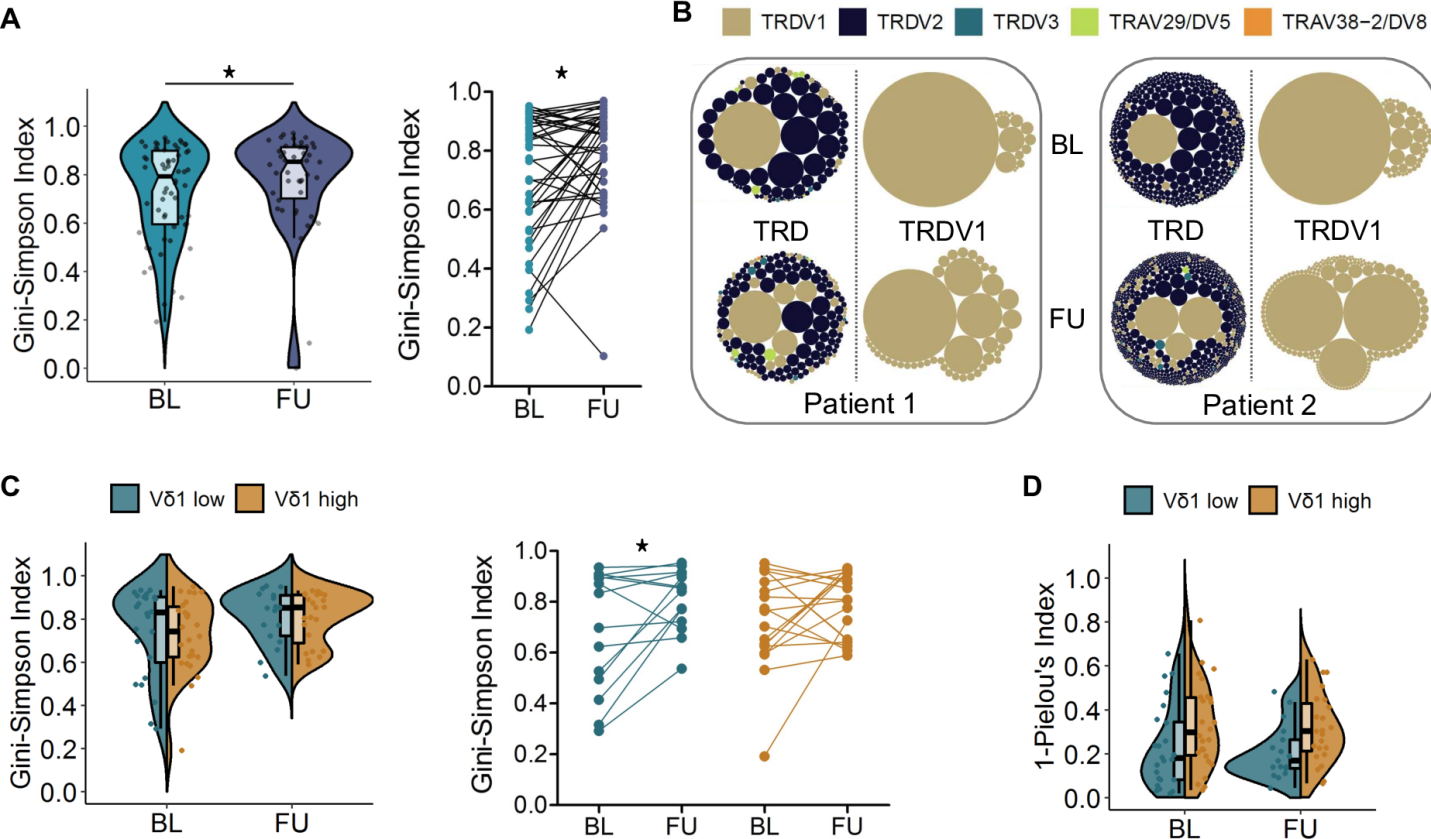
Diversity of the TRDV1 repertoire increases under anti-PD-1 therapy. (**A**) TRDV1 repertoire diversity in PBMCs from patients with melanoma at BL (n=60) and FU (n=50) represented by the Gini-Simpson Index. A higher index represents higher diversity. A significant increase in diversity was observed early under therapy, particularly in patients with low Gini-Simpson indices at baseline, indicating the appearance of new, or low frequent clones. (**B**) Tree maps illustrating the TRD and TRDV1 repertoire composition of two patients with a high increase of the Gini-Simpson Index under therapy. (**C**) Comparison of TRDV1 repertoire diversity and changes therein in the Vδ1^low^ (BL: n=24; FU: n=17) and Vδ1^high^ (BL: n=26; FU: n=23) group. (**D**) Clonality (1-Pielou’s index) of the TRDV1 repertoire in the Vδ1^low^ (BL: n=24; FU: n=17) and Vδ1^high^ (BL: n=26; FU: n=23) group. A higher index indicates greater clonality. This was observed in Vδ1^high^ patients, whose Vδ1 populations are characterized by late differentiated and senescent-like phenotypes. Phenotypic data allowing assignment to the Vδ1^low^ or Vδ1^high^ group were not available for all patients. Groups compared by Mann-Whitney U test, for paired analysis by Wilcoxon matched pairs signed-rank test. *p<0.05. BL, baseline; FU, follow-up; PBMC, peripheral blood mononuclear cell; PD-1, programmed cell death receptor 1.

Sorted peripheral blood γδ T cells were bulk sequenced in order to investigate the composition of the TCR δ-chain (TRD) repertoire. Diversity of the TRDV1 repertoire was evaluated using the Gini-Simpson Index, with a higher index representing higher diversity. We observed a significant increase in diversity early under therapy, especially in patients with low Gini-Simpson indices before the start of therapy (figure 4A). A higher Gini-Simpson Index is associated with a more equitable representation of clonotypes, as illustrated by the treemaps showing two patients with a strong increase in repertoire diversity in figure 4B, indicating an ICB-induced polyclonal expansion of Vδ1 T cells. No public Vδ1 sequences or sequence motifs were identified in this patient cohort, in agreement with the high magnitude of junctional combinations of the TCR δ gene.^50 51^ Even though Vδ2 T cells express PD-1 and expression declined under therapy (figure 1A), no changes were detected with regard to TRDV2 repertoire diversity online supplemental figure S16, underlining the divergent biology of this innate-like γδ T-cell subset. Comparison of the Vδ1^low^ to the Vδ1^high^ group revealed no differences in TRDV1 diversity, neither at BL nor at FU (figure 4C). However, a significant increase in the Gini-Simpson Index under therapy was seen only in the Vδ1^low^ group, consistent with the observed lower prevalence of late-differentiated or replicative-senescent cells, while the Vδ1^high^ group displayed alterations in both directions (figure 4C). Analysis of CDR3 length distribution revealed no differences between patients and HD and no influence of anti-PD-1 therapy online supplemental figure S17A. Furthermore, the Vδ1^low^ and Vδ1^high^ group presented a similar pattern at BL and FU online supplemental figure S17B. Skewing or reshaping of the CDR3 spectratype would be characteristic of an oligoclonal expansion. The 1-Pielou’s index was used to assess clonality of the TRDV1 repertoire. We noted a trend towards a higher clonality in the Vδ1^high^ group at BL (p=0.056) as well as at FU (p=0.055) (figure 4D), hinting at a higher prevalence of expanded clones in this patient group. Comparison of BL and FU samples showed mixed alterations in both groups online supplemental figure S16C.

### TCR repertoire of tumor-infiltrating γδ T cells overlaps with peripheral blood

**Figure 5:**
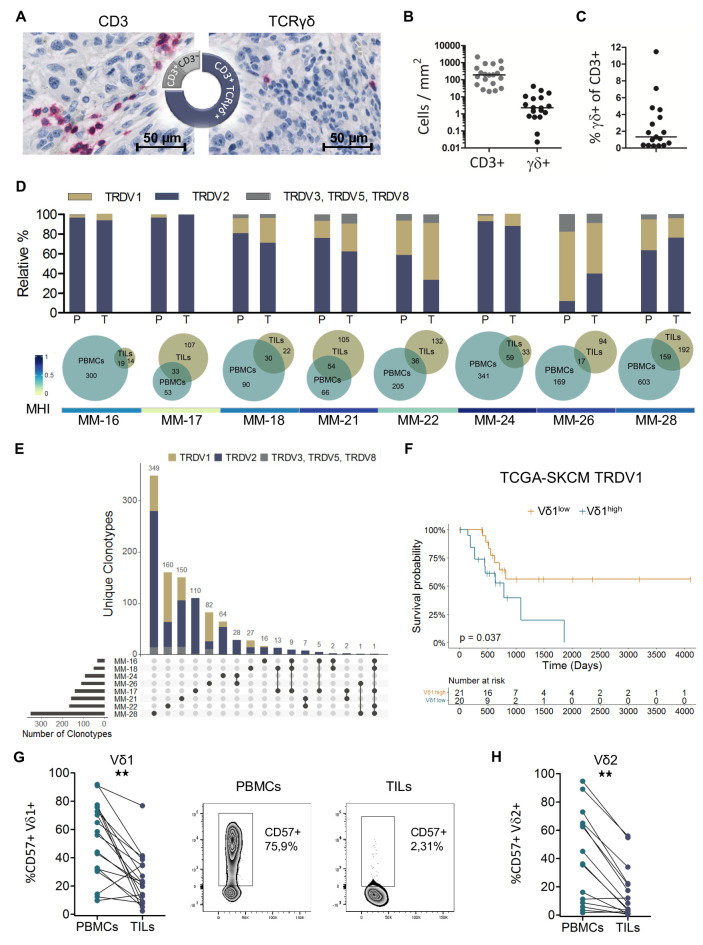
Melanoma infiltrating Vδ1 T cells are positively associated with survival and a lower CD57 frequency. (**A**) Representative IHC staining of CD3 and TCRγδ. Pie chart depicts the proportion of metastatic tumor samples showing infiltration with γδ T cells, non-γδ T cells only or no T-cell infiltration (n=24). (**B**) Number of CD3^+^ and TCRγδ^+^ cells per area and (**C**) Percentage of γδ T cells among all T cells estimated from serial sections of the observed 24 patients. (**D**) Relative frequencies of TRDV1, TRDV2 and TRDV3/TRDV5/TRDV8 clonotypes in paired samples of PBMCs and TILs. Proportional Venn diagrams illustrate the number of detected clonotypes and the proportion of overlapping clonotypes between PBMCs and TILs for each patient (n=8). The heatmap displays pairwise TRD repertoire similarity between PBMCs and TILs assessed using the MHI. (**E**) Clonotype intersections between tumor-infiltrating TCRγδ T cells of metastatic tumor samples. A vertical bar chart represents unique clonotypes and clonotype composition, and a horizontal bar chart shows the number of clonotypes per patient. Connected dots indicate shared clonotypes. (**F**) Overall survival of stage IIIc and IV patients from TCGA SKCM data set dichotomized based on median TRDV1 expression. Survival distribution compared by log-rank test (n=41). Frequency of CD57^+^ cells in paired PBMC and TIL samples for Vδ1 (**G**) and Vδ2 (**H**) T cells (n=18). Groups compared by Wilcoxon matched pairs signed-rank test. **p<0.01. IHC, immunohistochemistry; MHI, Morisita-Horn Index; PBMC, peripheral blood mononuclear cell; SKCM, skin cutaneous melanoma; TCGA, The Cancer Genome Atlas; TIL, tumor-infiltrating lymphocyte.

The intratumoral presence of γδ T cells in melanoma metastases was assessed by immunohistochemistry. Three-quarters of the studied samples showed γδ T-cell infiltration and in about half of the remainder no T cells were detected, while the rest was infiltrated by non-γδ T cells (figure 5A). Quantitative analysis revealed a low abundance of γδ T cells (figure 5B), which represented less than 2.0% of infiltrating T cells (figure 5C). Next, the TRD repertoire of paired PBMC and TIL samples was analyzed. TRDV2 sequences dominated in most patients, although two had a high abundance of TRDV1 clonotypes (figure 5D). Overall, the TRD distribution was similar between the peripheral blood and TILs in each individual patient. As illustrated by the Venn diagrams, clonotypes overlapped between these two compartments, implying an exchange between blood and tumor (figure 5D). The pairwise similarity between the PBMC and TIL TRD repertoire was assessed using the MHI with an index of 1 representing identical repertoires and 0 no overlap between clonotypes. The MHI was above 0.5, with two exceptions, and dominant clonotypes were shared, further supporting trafficking of γδ T cells between periphery and tumor. More in-depth analysis revealed that up to 28 clonotypes were shared between the TILs of two patients and some clonotypes were present in several patients. However, only TRDV2 CDR3 sequences were shared, while TRDV1 and TRDV3/TRDV5/TRDV8 clonotypes were private (figure 5E). The TRDV2 CDR3 amino acid sequence “CACDTLGDTDKLIF” was detected in the TILs of five patients and, with one exception, was also present in the corresponding PBMC samples. This public clonotype with no N additions has been described previously in the peripheral blood by Papadopoulou *et al* in 9 out of 14 samples from 10-week-old donors,^52^ by Kakimi *et al* in 2 out of 5 patients with non-small cell lung cancer^53^ and by Deng *et al* in 76 out of 89 individuals including newborns, infants and adults.^54^

### Intratumoral Vδ1 T cells are positively associated with survival and a lower prevalence of replicative-senescent cells

Due to the limited availability of tumor samples, we sought to expand our analyses and assess the role of Vδ1 T cells in the tumor using a public dataset from (TCGA) SKCM project. Following Wu *et al*,^55^ TRDV1 expression was used as proxy for Vδ1 T cells and patients were stratified based on median expression. In contrast to peripheral blood, high TRDV1 expression in the tumor was significantly associated with favorable OS in stage IIIc and IV patients (figure 5F). This potential paradox could be resolved by investigating CD57 expression on paired peripheral blood and tumor samples (figure 5G). For the Vδ1 as well as for the Vδ2 (figure 5H) subset, we observed significantly lower percentages of CD57^+^ cells in the tumor, indicating a different subset composition and a higher proliferative potential in the tumor compartment.

## Discussion

Despite recent advances in cancer immunotherapy, prognosis of patients with melanoma with distant metastases remains poor and only a minority of patients achieves a durable response. γδ T cells mediate mostly antitumor and more rarely protumor functions and have been associated with both favorable and unfavorable clinical outcome in different cancer entities.^23 56^ By comparing the periphery and tumor, we extend previous observations and resolve the apparent paradox of a negative survival association of the Vδ1 frequency in the blood, while a higher abundance of Vδ1 T cells in the tumor is beneficial.

PD-1 blockade reinvigorates exhausted CD8 T cells; this mechanism of action likely extends to other subsets and is not restricted to the tumor microenvironment, but depends on immunomodulation of the peripheral blood and lymphoid compartment.^57^ T-cell exhaustion occurs in adaptation to persistent stimulation and is a dynamic process whereby long-lived PD-1 ^intermediate^ progenitor exhausted cells with low cytotoxicity give rise to short-lived terminally exhausted PD-1 ^high^ cells. The latter subset is characterized by high cytotoxic activity and the expression of multiple inhibitory receptors including PD-1, T cell immunoglobulin and mucin domain-containing protein 3 (TIM-3) and CTLA-4. While progenitor exhausted cells can be reinvigorated by PD-1 blockade, terminally exhausted cells are considered unresponsive^58^ and patients with melanoma with higher proportions of progenitor exhausted cells in pre-therapy biopsies experienced a longer OS under combination therapy.^59^ Our CyTOF experiments did not indicate co-expression of inhibitory receptors, especially not in cluster 1, which distinguished the Vδ1^high^ group. Moreover, the percentage of PD-1^+^ Vδ1 T cells was similar in the two patient groups. Therefore, the level of exhaustion does not seem to differ and is unlikely to account for the differences in OS under anti-PD-1 immunotherapy. Alongside exhaustion, T-cell senescence is another crucial state to consider in the context of dysfunctional tumor immunity.^60^ High levels of senescent peripheral T cells have been associated with poor OS in patients undergoing chemoradiotherapy, PD-(L)1 blockade or cancer vaccination in various solid cancer entities.^61^ T-cell senescence is marked by shortened telomeres and a loss of proliferative capacity. Characteristic phenotypic alterations include downregulation of the costimulatory receptors CD27 and CD28, and high expression of CD57 and KLRG1.^60^ CD57 has been linked to replicative senescence and likely has the same implication for γδ T cells, since CD57^+^ γδ T cells possess shorter telomeres and have low proliferative potential.^62^ In addition to higher proportions of cells with a TEMRA-like phenotype, we detected higher percentages of CD57^+^ cells in the Vδ1^high^group and an overall diminished proliferation of γδ T cells in all observed patients with melanoma. Furthermore, senescent T cells show an upregulation of a variety of activating and inhibitory NK cell receptors,^63 64^ increased expression of granzyme B and perforin, and secrete high levels of pro-inflammatory cytokines.^65^ Cluster 1, dominating the Vδ1^high^ group, displayed high expression of the NK cell-related markers CD56, NKG2A and CD161 as well as high perforin levels. The cytokine expression profile was not different between the patient groups or in comparison to HD, though PMA/ionomycin might not be optimal for assessing such differences since these stimuli bypass the TCR. Together, this implies an enrichment of senescence-associated features in patients with high Vδ1 frequencies. Being aware of the limitation that there are no mutually exclusive and inclusive phenotypic markers defining exhausted or senescent T cells, we propose the term “late-differentiated senescent-like” phenotype.

CD28 has been described as the primary target for PD-1-mediated T-cell suppression^66^ and CD28^+^ CD8 T cells were the predominant subset proliferating under PD-1 blockade in patients with lung cancer.^67^ Nonetheless, recent work indicates that CD28 is not required for responsiveness but serves as a marker for cells responsive to PD-1 blockade.^68^ Vδ1^high^ patients are characterized by increased levels of late-differentiated senescent-like cells that have lost CD28 expression. Expression of NK-related markers hints at an NK cell-like functionality, which could be advantageous due to a broadening of protective functions.^63^ However, loss of CD28 likely marks these cells as unresponsive to anti-PD-1 therapy, so high percentages of these cells might be disadvantageous in the current immunotherapy setting. For example, in advanced non-small cell lung cancer, elevated frequencies of CD28^−^CD57^+^KLRG1^+^ CD8 T cells were associated with poor OS in patients receiving anti-PD-(L)1 therapy.^69^

In patients with melanoma under anti-PD-1 therapy, we observed a decrease in the percentage of CD57^+^ Vδ1 T cells, an increase in TRDV1 repertoire diversity, but no skewing of the CDR3 spectratype, suggesting polyclonal expansions of less-abundant CD57^−^ Vδ1 clones. Since TRDV1 repertoires would be expected to remain stable over the investigated period (median of 43 days),^43^ we conclude that a direct or indirect therapy-induced change in TCR repertoires was responsible. This increase in repertoire diversity was only significant in the Vδ1^low^ group, in line with a reduced number of PD-1 blockade-responsive cells in Vδ1^high^ patients. Clonality tended to be higher in the Vδ1^high^ group, which displayed elevated frequencies of CD57^+^ cells, consistent with oligoclonal expansions predominantly described in the CD57^+^ CD8 T cells.^70^

IHC confirmed that γδ T cells infiltrate melanoma metastases^25 26 71^ and the substantial numbers of shared clonotypes between TILs and PBMCs as well as the degree of TRD repertoire similarity support an ongoing exchange between the peripheral blood and tumor. Within the tumor-infiltrating γδ T cells, we detected several shared Vδ2 clonotypes, including the public clonotype “CACDTLGDTDKLIF” previously described by others.^52^,^54^ In contrast, no Vδ1 clonotypes were shared between the eight studied patients, similar to a recent report by Wu *et al*.^72^ In choroidal melanomas, the presence of Vδ1 T cells in the tumor correlated positively with survival.^73^ Higher numbers of effector memory Vδ1 T cells in the tumor as well as tissue-resident memory Vδ1 T cells in non-malignant lung tissue were associated with a significantly longer relapse-free survival in resected non-small cell lung cancer.^55^ Furthermore, reanalysis of RNA sequencing data from the INSPIRE trial,^74^ evaluating the effect of pembrolizumab treatment across a variety of advanced solid cancers including melanoma, showed that higher than median TRDV1 expression in tumor biopsies was linked to prolonged OS.^55^ These reports are in line with our analysis of the TCGA SKCM dataset showing a favorable OS in late-stage patients with above median TRDV1 expression. The significantly lower percentage of CD57^+^ cells within the Vδ1 population in TILs compared with peripheral blood implies a lower prevalence of the late-differentiated senescent-like phenotype in the tumor. In patients with higher frequencies of this phenotype in the blood, the Vδ1 compartment might have a limited potential to expand in the tumor microenvironment and therefore poorer chances of mounting or orchestrating an efficient antitumor response.

With regard to the Vδ2 subset, our data revealed no association with clinical outcome and no alterations under anti-PD-1 therapy. This suggests a minor role of this subset in the studied setting, although it is clear that Vδ2 T cells are able to exert antitumor effector functions.^23 75^ However, Vδ2 T cells of patients with melanoma showed an altered phenotype with increased levels of CD57^+^ cells and an impaired functionality in response to phosphoantigen stimulation compared with HD, confirming findings from previously published studies in a more comprehensive and detailed manner.^76^,^78^

The main focus of γδ T-cell immunotherapy has been on targeting Vδ2 T cells either through stimulation with aminobisphosphonates or in an adoptive transfer setting, although more recently the focus has shifted to the Vδ1 subset.^79^,^81^ Human melanoma-derived Vδ1 T-cell lines mediated cytotoxicity against a melanoma cell line,^82^ and Vδ1 T cells have been shown to traffic to the tumor site and reduce tumor growth in a human melanoma xenograft SCID mouse model.^83^ The results of clinical trials investigating Vδ2 T cells have been disappointing so far.^81^ Since Vδ1 T cells are considered naturally tissue resident, they might be better equipped to traffic to and cope with the hardships of the tumor microenvironment. In addition, based on their MHC independence, γδ T cells are being considered in allogeneic transfer settings even as off-the-shelf products.^84^ There are several ongoing clinical trials and an increasing number of companies using Vδ1 T cells for adoptive transfer or antibody-based strategies to target solid cancer.^85^ Our data, linking Vδ1 T cells to survival outcome and demonstrating a high prevalence of a presumably ICB-unresponsive phenotype in poor survivors, encourage these approaches. However, with regard to adoptive cell therapy (chimeric antigen receptor/engineered TCR/Delta One T-cell therapy^86 87^) the phenotype of the expanded product should be carefully evaluated, because high proportions of late-differentiated senescent-like cells are likely disadvantageous. Moreover, this supports allogeneic approaches, since in the autologous setting one is restricted by the patients’ phenotypic profile. Furthermore, a better understanding of the mechanisms underlying senescence might allow targeting of this dysfunctional state.^61^

A limitation of our study is that knowledge of T-cell exhaustion and senescence stems from investigation of αβ T cells and it remains unclear to which extent these concepts apply to γδ T cells. Nevertheless, there is increasing evidence that Vδ1 T cells follow an adaptive differentiation process analogous to CD8 T cells, ^18 88^ indicating parallels in the underlying biology. Furthermore, the precise therapeutic setting of the patients in the TCGA SKCM dataset is unknown and might not be directly comparable to our patient cohorts. Thus, a larger number of tumor samples and phenotypic markers, ideally in situ, would need to be examined to explore associations with clinical outcome related to anti-PD-1 therapy and how the abundance and composition of tumor-infiltrating γδ T cells relates to the phenotypic profile in peripheral blood. However, recent work by Davies *et al* shows that high intratumoral expression of TRDV1 is linked to superior progression-free survival of patients with melanoma receiving anti-PD-1 or anti-PD-L1 therapy. ^89^

In conclusion, we report a robust association of high peripheral Vδ1 frequencies and poor OS of patients with late-stage melanoma undergoing PD-1 blockade. These results are similar to our previous observations in patients receiving ipilimumab.^32^ The present study extends these findings to reveal that these Vδ1 T-cell populations are enriched in cells exhibiting a late-differentiated senescent-like phenotype. Such cells may be dysfunctional and resistant to reactivation by anti-PD-1 therapy. In contrast, enrichment of Vδ1 T cells in the tumor itself was found to be associated with prolonged OS. The majority of tumor-infiltrating Vδ1 T cells did not exhibit a senescent-like phenotype. Thus, we hypothesize that the tumor microenvironment in patients responding to ICB was more conducive to selecting functional, presumably therapy-responsive clones. Consistent with this, we show an alteration of the TRDV1 repertoire composition early under therapy, suggesting ICB-related polyclonal expansions. Altogether, our data support the exploitation of Vδ1 T cells for novel cancer immunotherapy approaches using T-cell engagers or adoptive cell therapy as a promising avenue.

## Supplementary material

10.1136/jitc-2024-011224 online supplemental figure 1

10.1136/jitc-2024-011224 online supplemental file 1

## Data Availability

Data are available upon reasonable request.
