# Supplementary figures and images for: Vδ1 T-cell subset appears to be responsive to PD-1 blockade therapy and is associated with survival in melanoma
Source: J Immunother Cancer. 2026 Jan 20;14(1):e011224. doi: 10.1136/jitc-2024-011224 (PMC12820842; doi:10.1136/jitc-2024-011224)

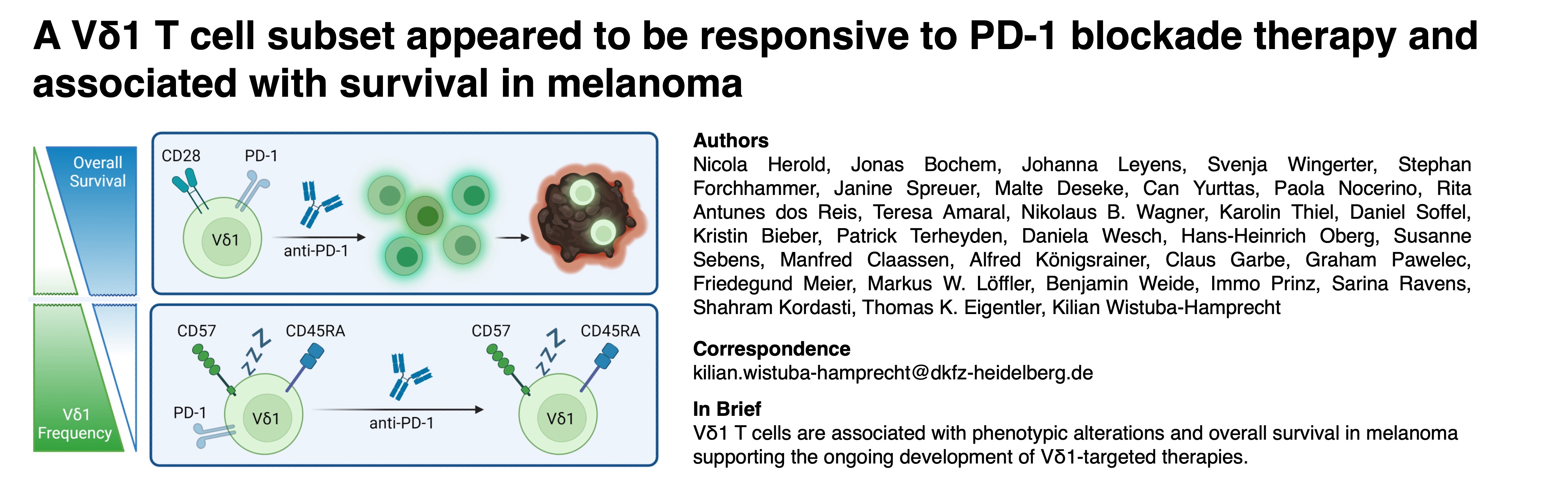

Supplement: online supplemental figure 1 [file jitc-14-1-s001.jpg]
