# Supplementary material for: Vδ1 T-cell subset appears to be responsive to PD-1 blockade therapy and is associated with survival in melanoma
Source: J Immunother Cancer. 2026 Jan 20;14(1):e011224. doi: 10.1136/jitc-2024-011224 (PMC12820842; doi:10.1136/jitc-2024-011224)
Supplement: online supplemental file 1 [file jitc-14-1-s002.pdf]

Supplementary Figures

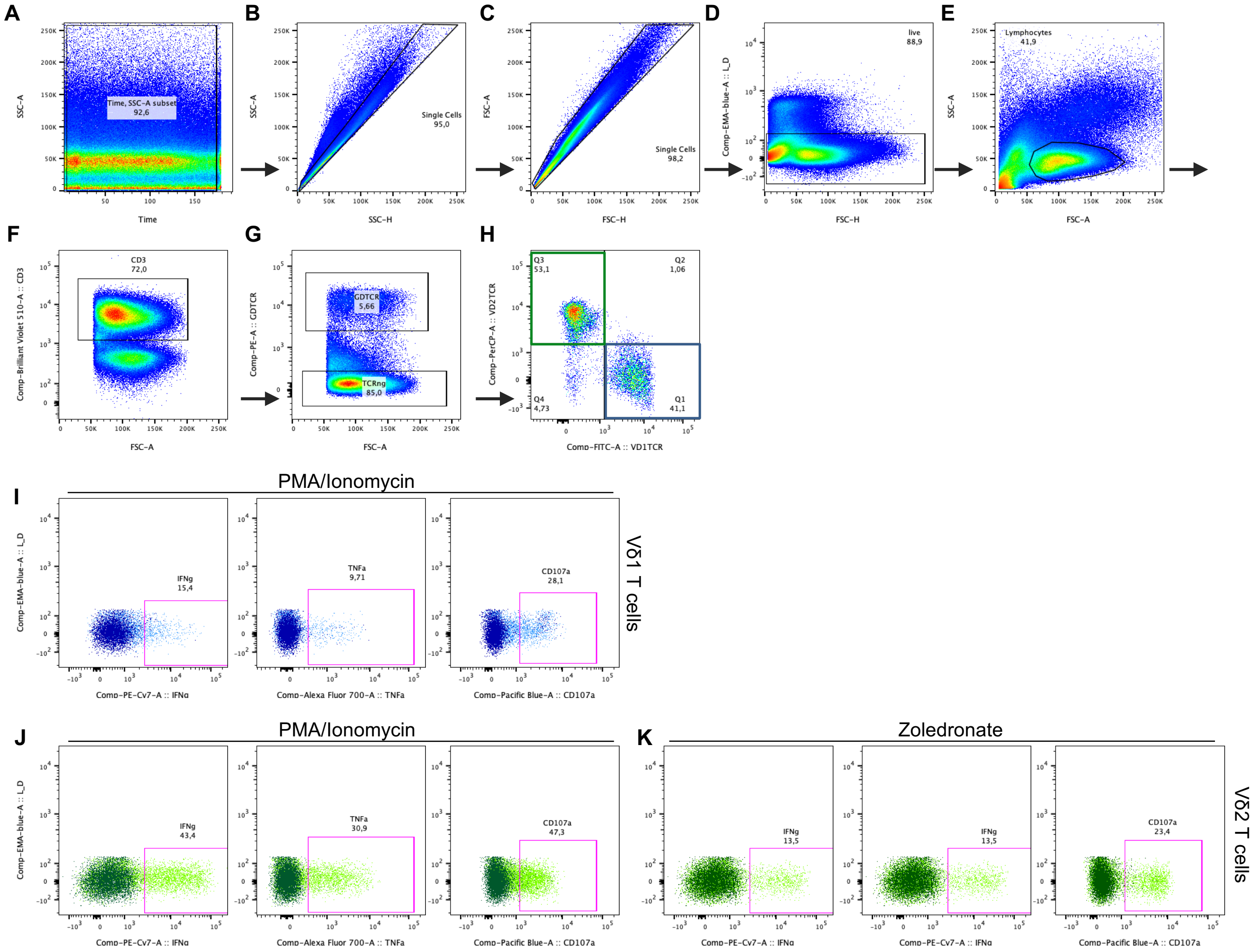

Fig. S1 Gating strategy for functional analysis.
(**A**) Gating started with a time gate to monitor potential fluctuations in the pressure system and exclude cross-contamination from the previous sample. (**B**)(**C**) Singlets were selected and (**D**) dead cells excluded, followed by (**E**) lymphocyte gating. (**F**) CD3+, (**G**) TCRγδ+ cells were subdivided into (**H**) Vδ1+ and Vδ2+ cells. Expression of functional markers (IFNγ, TNF & CD107a) was quantified relative to the not stimulated sample (dark versus light coloring in the overlayed dot plots) for PMA/Ionomycin stimulation of Vδ1+ **(I)** Vδ2+ T cells (**J**) and for zoledronate stimulation of Vδ2 T cells (**K**).

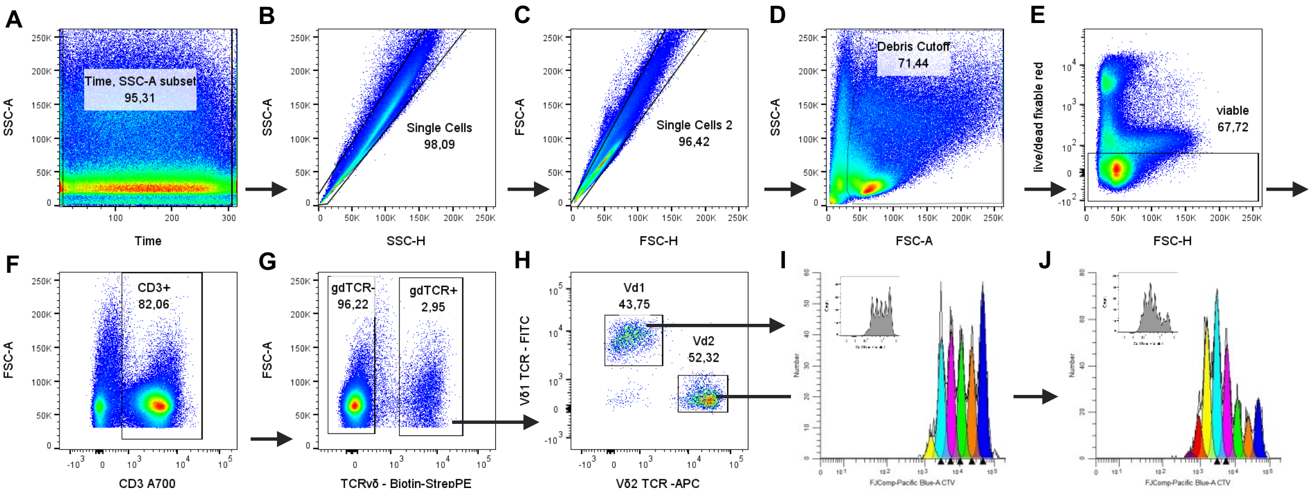

Fig. S2 Gating strategy for proliferation analysis.
(**A**) Gating started with a time gate to monitor potential fluctuations in the pressure system and exclude cross-contamination from the previous sample. (**B**)(**C**) Singlets were selected. (**D**) Debris was excluded and no lymphocyte gate applied to avoid bias, because proliferating cells deviate in size and granularity from non-proliferating lymphocytes. (**E**) Dead cells were excluded. (**F**) CD3+, (**G**) TCRγδ+ cells were subdivided into (**H**) Vδ1+ and Vδ2+ cells. (**I**)(**J**) Proliferation of both subsets was examined using the ModFit proliferation analysis tool.

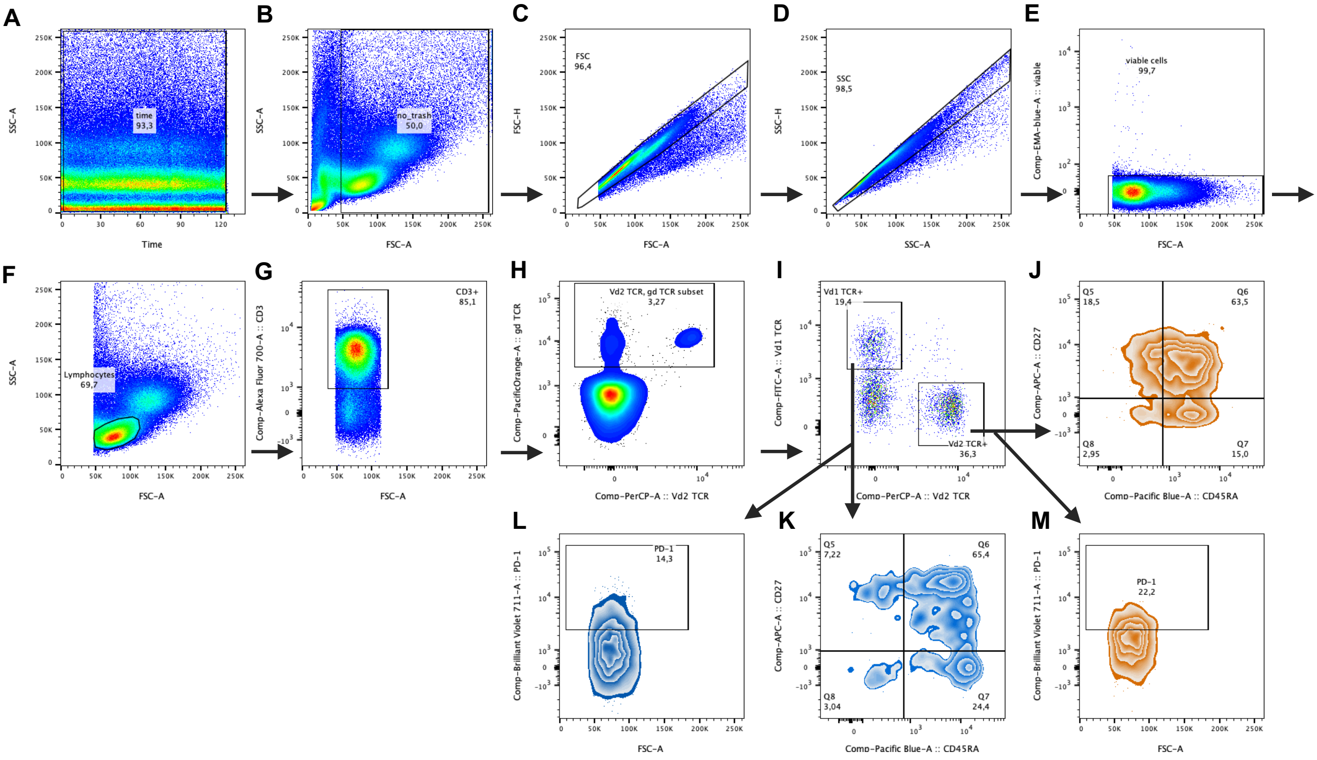

Fig. S3 Gating strategy differentiation panel.
(**A**) Gating started with a time gate to monitor potential fluctuations in the pressure system and exclude cross-contamination from the previous sample. **(B)** Debris was excluded. (**C**)(**D**) Singlets were selected and (**E**) dead cells excluded, followed by (**F**) lymphocyte gating. (**G**) CD3+, (**H**) TCRγδ+ cells were subdivided into (**I**) Vδ1+ and Vδ2+ T cells. (**J**)(**K)** Differentiation subsets were gated based on differential expression of CD27 and CD45RA. (**L**)(**M**) PD-1 expression was quantified on Vδ1+ and Vδ2+ T cells.

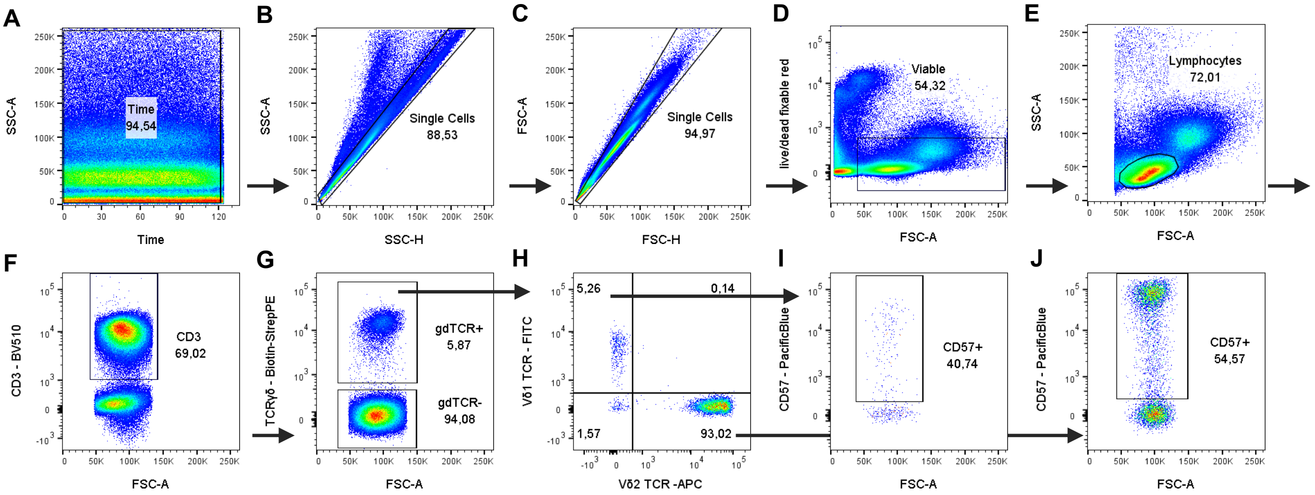

Fig. S4 Gating strategy phenotypic panel.
(**A**) Gating started with a time gate to monitor potential fluctuations in the pressure system and exclude cross-contamination from the previous sample. (**B**)(**C**) Singlets were selected and (**D**) dead cells excluded, followed by (**E**) lymphocyte gating. (**F**) CD3+, (**G**) TCRγδ+ cells were subdivided into (**H**) Vδ1+ and Vδ2+ cells. (**I**)(**J**) CD57 expression on both subsets was examined.

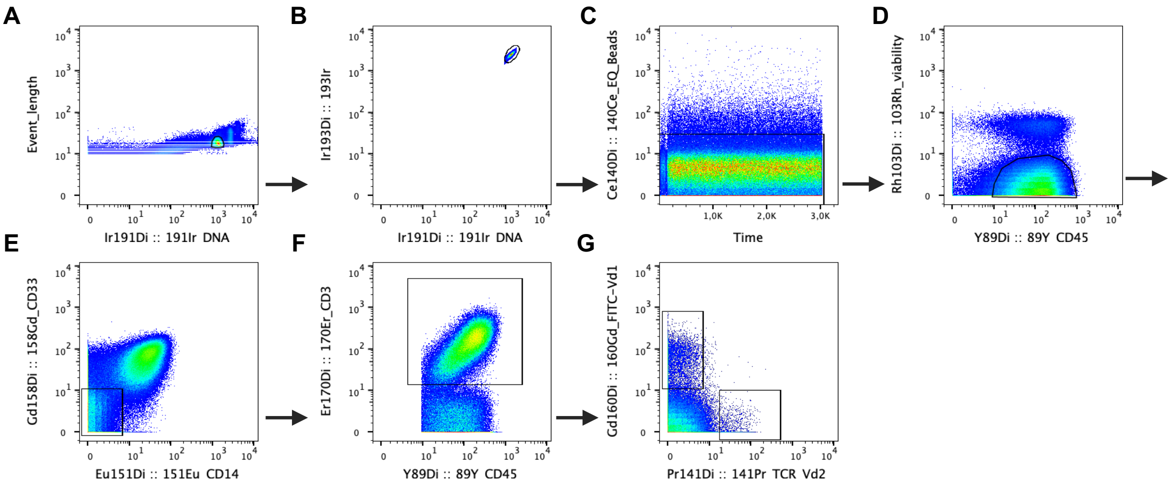

Fig. S5 Gating strategy for mass cytometry.
(**A**) Intact cells were identified based on event length and Ir-191, (**B**) followed by Ir193 versus Ir191. (**C**) Exclusion of EQ beads. (**D**) Viable, rhodium negative CD45+ cells were selected. (**E**) Cells expressing CD33 and/or CD14 were excluded. (**F**) CD3+ cells were subdivided into (**G**) Vδ1+ and Vδ2+ cells.

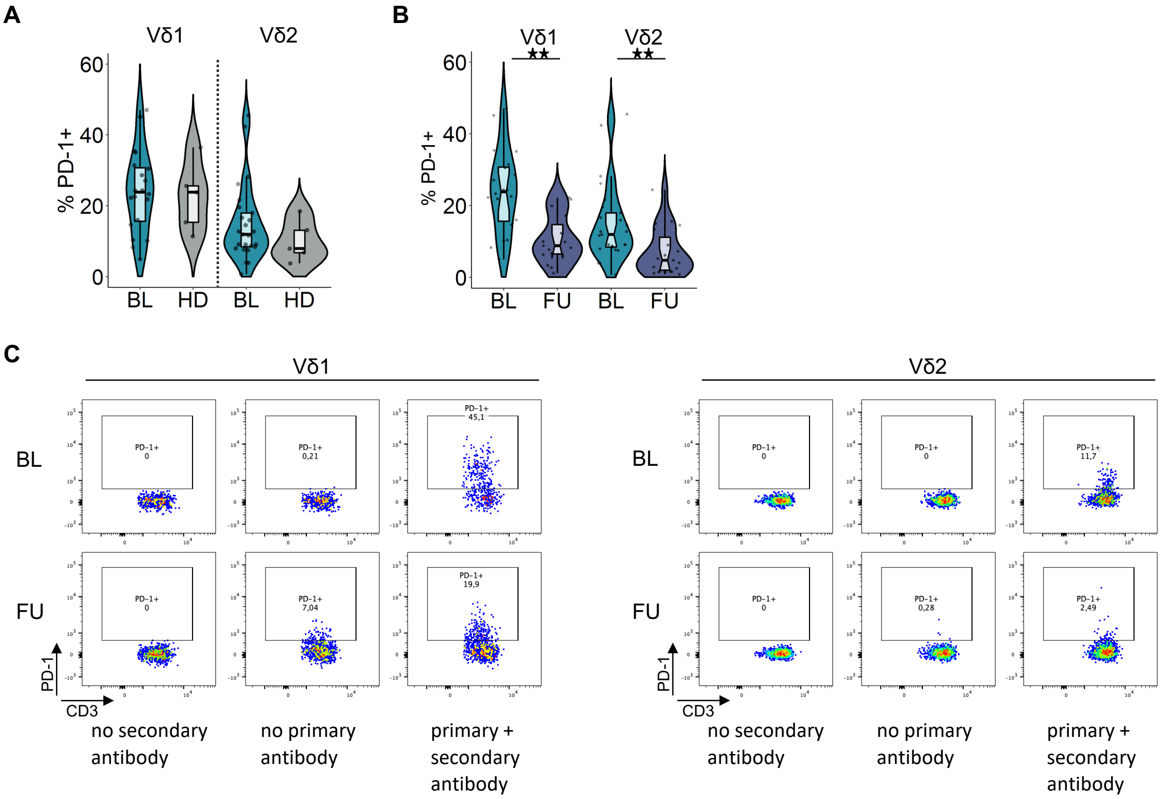

Fig. S6 PD-1 expression on γδ T cells.
(A) Percentage of PD-1-expressing Vδ1 and Vδ2 T cells in patients before the start of therapy (BL) and healthy donors (HD). **(B)** Percentage of PD-1-expressing Vδ1 and Vδ2 T cells in patients before the start of therapy (BL) and under therapy (FU). Paired comparison using the Wilcoxon matched pairs signed rank test **P < 0.01 **(C)** Analysis of PD-1 expression in patients at follow-up (FU) under anti-PD-1 therapy. Flow cytometric detection of therapeutic antibody (Pembrolizumab) on Vδ1 and Vδ2 T cells using an anti-IgG4 PE antibody.

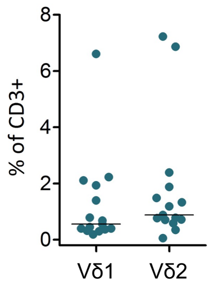

Fig. S7 Frequency of Vδ1 and Vδ2 T cells within all T cells.
Percentage of CD3+ cells expressing Vδ1 or Vδ2 in melanoma patients before the start of therapy. Horizontal lines denote the median.

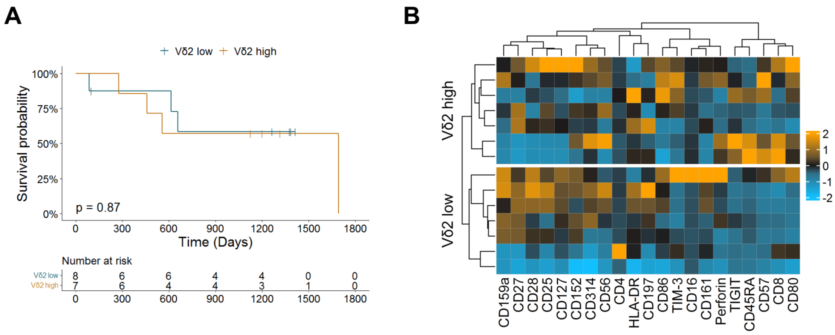

Fig. S8 Vδ2 T cell frequencies are not associated with survival.
(A) Overall survival of late-stage melanoma patients dichotomized based on the BL median Vδ2 frequency (0.88 %) determined by mass cytometry. Survival distribution compared by log rank rest. **(B)** Heat map of phenotypic marker expression on Vδ2 T cells. Each row represents the BL sample of one patient, heatmaps are divided based on median Vδ2 frequency. Marker expression was arcsinh-transformed and normalized to a mean of 0 and a standard deviation of 1. Relative overexpression indicated in orange, relative underexpression in blue.

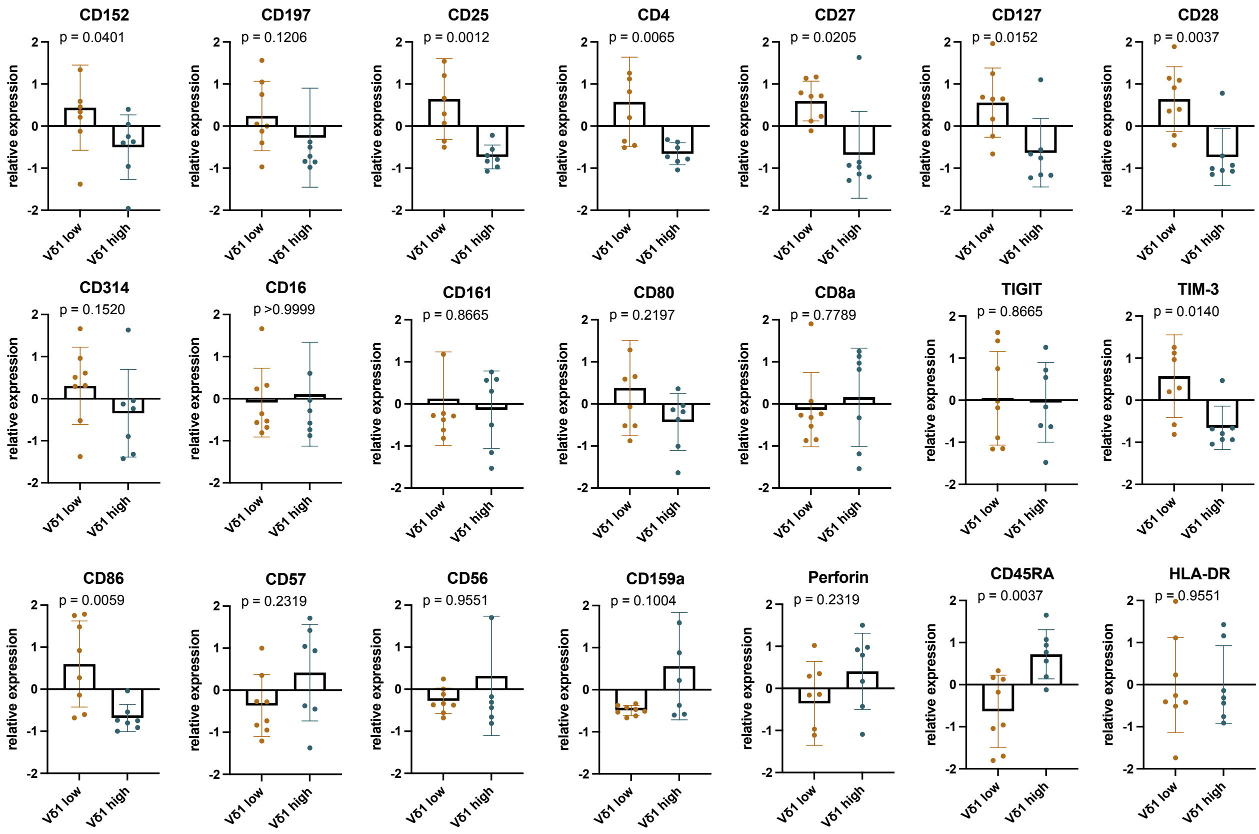

Fig. S9 Relative marker expression of Vδ1 T cells
Relative marker expression of Vδ1 T cells from patients in Cohort 1 prior to the initiation of ICB, as summarized in the heat map in Figure 1C. The cohort was dichotomized based on the median Vδ1 T cell frequency. Individual plots display marker expression values, which were arcsinh-transformed and standardized (mean = 0, standard deviation = 1). Group comparisons were performed using the Mann–Whitney U test.

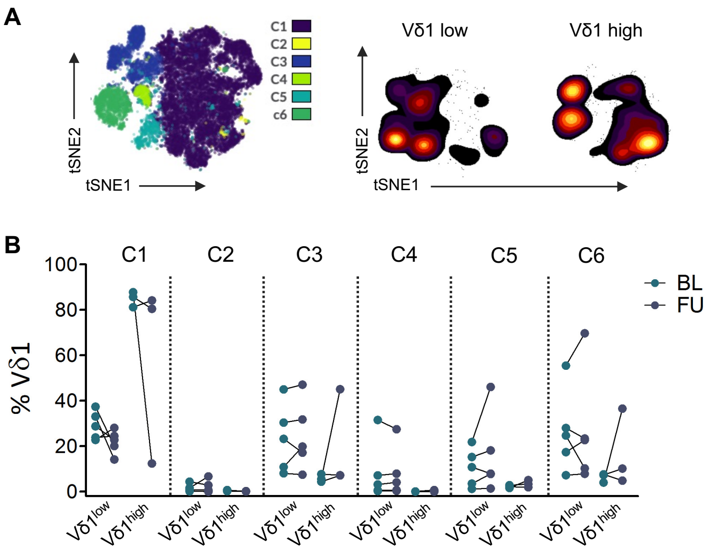

Fig. S10 Changes in Vδ1 cluster frequencies under therapy.
(**A**) tSNE visualization of Vδ1 T cells from BL and FU samples. Clusters were annotated using FlowSOM. Smoothed contour plots show FU samples of the patient groups with lower and higher than median Vδ1 frequencies at BL. **(B)** Change of the Vδ1 FlowSOM cluster frequencies from BL to FU for patients with high and low Vδ1 frequencies.

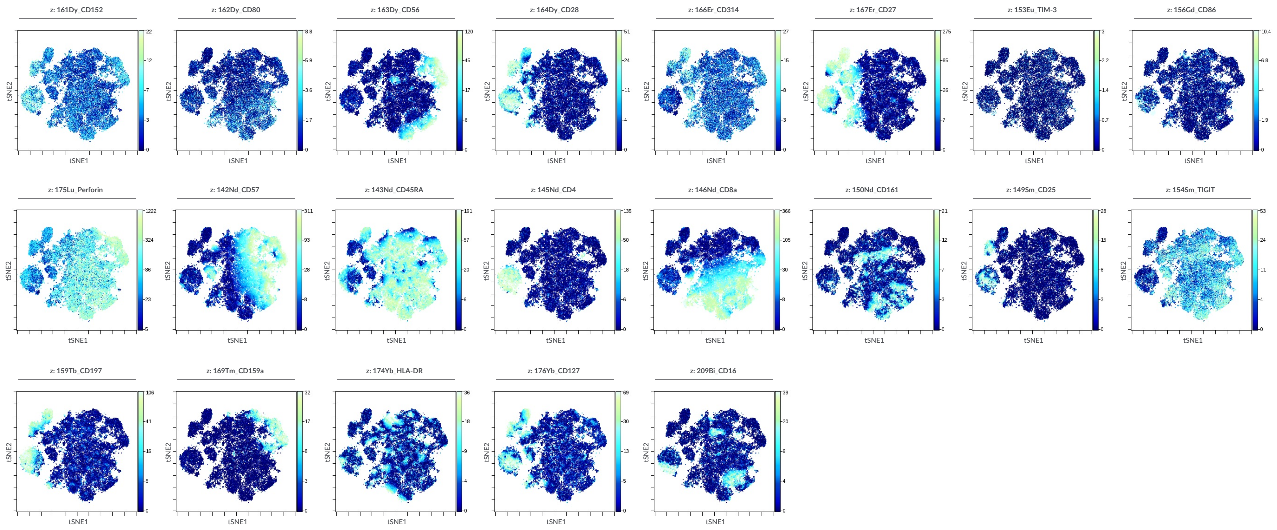

Fig. S11 Expression profile of Vδ1 T cells determined by mass cytometry.
Distribution of relative arcsinh-transformed marker expression intensities for Vδ1 T cells in all investigated samples projected on the tSNE visualization.

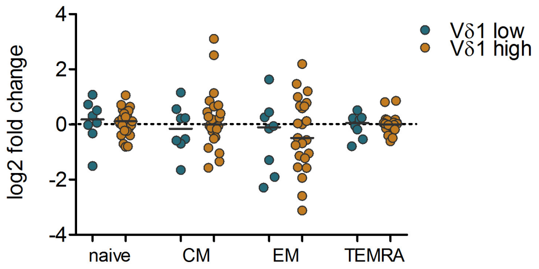

Fig. S12 Fold change in Vδ1 memory differentiation profile.
Log2 fold change between BL and FU for naive, central memory (CM), effector memory (EM) and TEMRA Vδ1 T cells for patients with high and low Vδ1 frequencies.

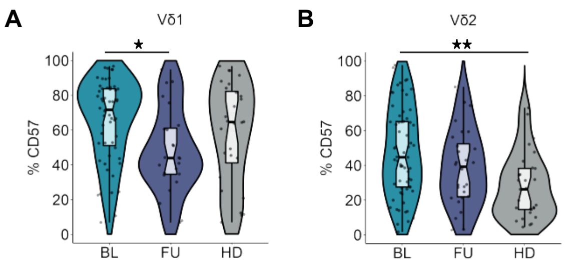

Fig. S13 CD57 expression in patients and healthy donors.
(A) Expression of CD57 on Vδ1 and **(B)** Vδ2 T cells in patients at BL and FU and in healthy donors. Groups compared by Man-Whitney-U test. *P < 0.05, **P < 0.01.

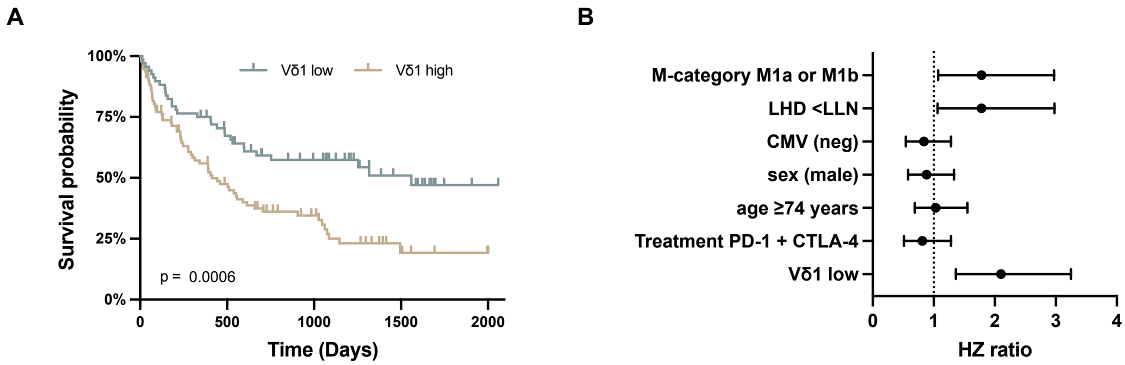

Fig. S 14 Combined analyses of cohort 2 and 3

(A) Overall survival of late-stage melanoma patients in combined analyses of cohort 2 and 3. Patients were dichotomized based on the predetermined Vδ1 cutoff frequency of 0.56% amongst all T cells (cohort 1). Survival distribution compared by log rank rest. (B) Forrest plot summarizing Hazard ratios for potentially confounding factors for survival analyses. The results of the underlying multivariate analysis are summarized in Table S2.

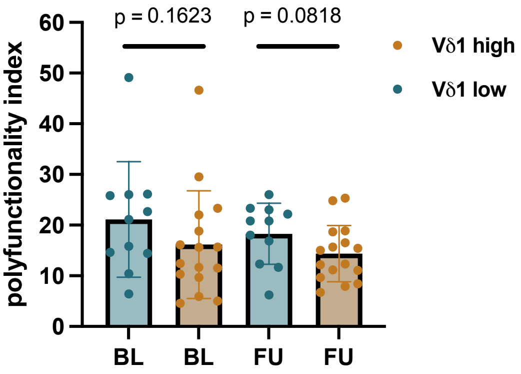

Fig. S15 Polyfunctionality index

Heatmap visualizing the polyfunctionality index (CD107a, IFN-γ, TNF) for Vδ1 T cells at BL and FU. Each dot represents one patient sample. Group comparisons were performed using the Mann–Whitney U test.

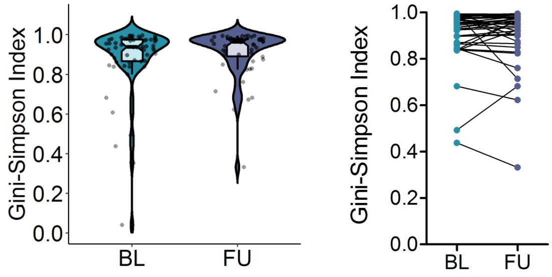

Fig. S16 Diversity of the TRDV2 repertoire.
TRDV2 repertoire diversity in PBMCs from melanoma patients at BL and FU represented by the Gini-Simpson index.

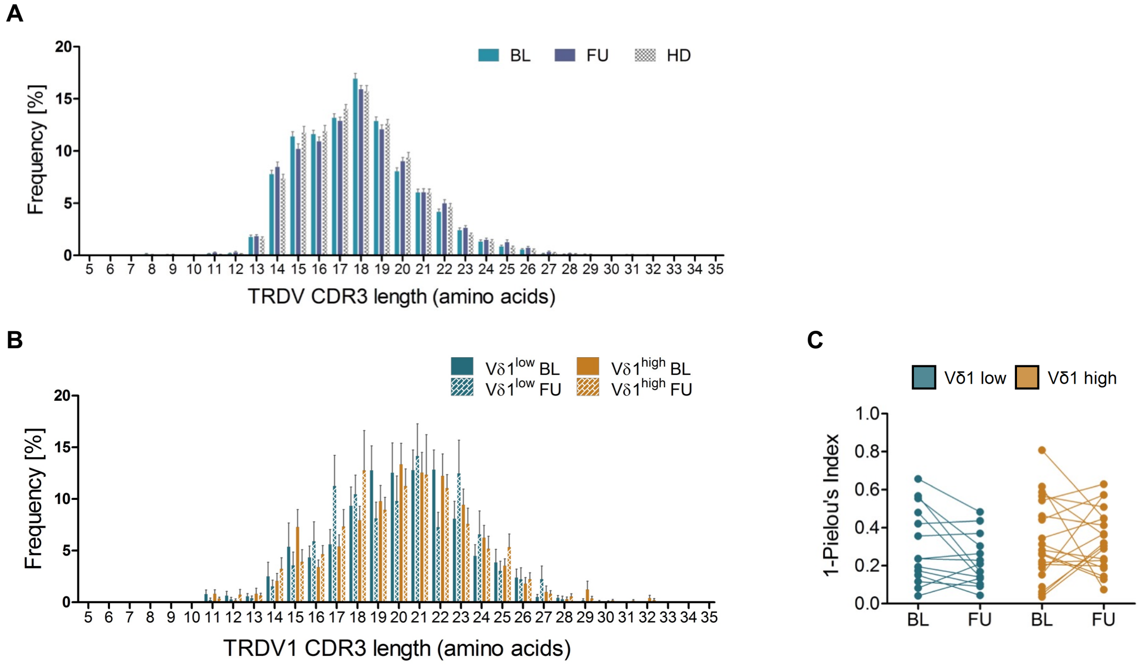

Fig. S17 Composition of the TRDV1 repertoire.
(**A**) Comparison of the TRDV repertoire CDR3 length distribution between patients at BL and FU and healthy donors. (**B**) Comparison of the TRDV1 repertoire CDR3 length distribution between BL and FU and the Vδ1^{low} and Vδ1^{high} group. (**C**) Changes in TRDV1 repertoire clonality (1-Pielou‘s Index) under therapy in the Vδ1^{low} and Vδ1^{high} group. Bars indicate mean, whiskers standard error of the mean.

Table S1: Patients characteristics

Factor	Category	Cohort 1 “CyTOF” n = 15		Cohort 2 “Differentiation” n = 91		Cohort 3 “CD57” n = 70	
		n	%	n	%	n	%
Clinical Site	Tübingen	15	100	63	69.2	51	72.9
	Dresden	0	0	17	18.7	16	22.9
	Lübeck	0	0	11	12.1	3	4.2
Treatment	anti-PD-1	15	100	91	100	25	35.7
	anti-PD-1 + anti-CTLA-4	0	0	0	0	45	64.3
Sex	Female	5	33.3	33	36.3	29	41.4
	Male	10	66.7	58	63.7	41	58.6
Stage	III	3	20.0	14	15.4	6	8.6
	IV	12	80.0	77	84.6	64	91.4
M category (AJCC)	M1a	2	13.3	12	13.2	6	8.6
	M1b	3	20.0	16	17.6	7	10.0
	M1c	9	60.0	53	58.2	53	75.7
	unknown	1	6.7	10	11.0	4	5.7
Age	Median	77		74		72	
	Range	65-82		36-94		23-89	

Table S2: Panel for mass cytometry

Specificity	Label	Clone	Manufacturer
TCR Vδ2	141Pr	B6	BioLegend
CD57	142Nd	HCD57	Fluidigm
CD45RA	143Nd	HI100	Fluidigm
CD4	145Nd	RPA-T4	Fluidigm
CD8a	146Nd	RPA-T8	Fluidigm
CD25	149Sm	2A3	Fluidigm
CD161	150Nd	HP-3G10	BioLegend
CD14	151Eu	M5E2	Fluidigm
TIM-3	153Eu	F38-2E2	Fluidigm
TIGIT	154Sm	MBSA43	Fluidigm
CD86	156Gd	IT2.2	Fluidigm
CD33	158Gd	WM53	Fluidigm
CD197	159Tb	G043H7	Fluidigm
anti-FITC	160Gd	FIT-22	Fluidigm
CD152*	161Dy	14D3	Fluidigm
CD80	162Dy	2D10.4	Fluidigm
CD56	163Dy	NCAM16.2	Fluidigm
CD28	164Dy	CD28.2	BioLegend
CD314	166Er	ON72	Fluidigm
CD27	167Er	L128	Fluidigm
CD159a	169Tm	Z199	Fluidigm
CD3	170Er	UCHT1	Fluidigm
HLA-DR	174Yb	L243	Fluidigm
Perforin*	175Lu	B-D48	Fluidigm
CD127	176Yb	A019D5	Fluidigm
CD16	209Bi	3G8	Fluidigm
CD45	89Y	HI30	Fluidigm
TCR Vδ2	FITC	TS8.2	Life Technologies

* Intracellular detection

Table S3: Univariate and multivariate analysis of potentially confounding factors

	feature	N	% dead	Univariate survival analysis					Multivariate Analysis	
				Median OS		OS				
				days	95% CI	HR	95% CI	p (log-rank test)	HR	95% CI
Age [years]	≥74	82	59.8	657	274-1040	1.03	0.69-1.55	0.875	0.99	0.64-1.53
	<74	79	57.0	557	239-875					
Sex	Male	99	60	532	328-736	0.88	0.58-1.33	0.546	0.99	0.63-1.57
	Female	62	56.5	1026	480-1572					
M-category*	M1a or M1b	41	46.3	1046	-	1.78	1.07-2.97	0.024	1.86	1.07-3.25
	M1c	106	63.2	487	355-618					
Treatment	Anti-PD1 + Anti-CTLA4	45	57.8	538	237-839	0.81	0.51-1.28	0.365	1.04	0.62-1.74
	Anti-PD1+	116	58.6	614	167-1061					
CMV**	seronegative	60	53.3	538	128-948	0.84	0.54-1.28	0.414	1.08	0.67-1.75
	seropositive	100	61.0	705	188-1222					
LDH	<ULLN	132	56.1	705	311-1098	1.78	1.06-2.98	0.027	1.52	0.88-2.64
	≥ULN	27	66.7	182	0-612					
VD1	Low	68	45.6	1559	-	2.10	1.36-3.25	<0.001	2.18	1.34-3.53
	high	93	67.7	413	249-577					

* M-category was unknown for 14 patients

** CMV serostatus was unknown for 1 patient

*** LDH was unknown for 2 patients
